# Gene expression profiling tests to guide adjuvant chemotherapy decisions in lymph node-positive early breast cancer: a systematic review

**DOI:** 10.1007/s10549-024-07596-0

**Published:** 2025-02-03

**Authors:** Katy Cooper, Gamze Nalbant, Munira Essat, Sue Harnan, Ruth Wong, Jean Hamilton, Uzma S. Asghar, Nicolò M. L. Battisti, Lynda Wyld, Paul Tappenden

**Affiliations:** 1 School of Medicine and Population Health, University of Sheffield, Regent Court, 30 Regent Street, Sheffield, S1 4DA UK; 2 Breast Unit, Department of Medicine, Oak Cancer Centre, The Royal Marsden NHS Foundation Trust, Sutton, SM2 5PT UK; 3 Breast Unit, Department of Medicine, The Royal Marsden NHS Foundation Trust, London, UK

**Keywords:** Systematic review, Gene expression profiling, Prognostic test, Breast neoplasms, Adjuvant chemotherapy

## Abstract

**Purpose:**

To systematically review the effectiveness of gene expression profiling tests to inform adjuvant chemotherapy decisions in people with hormone receptor-positive (HR+), lymph node-positive (LN+) breast cancer.

**Methods:**

This systematic review assessed the effectiveness of Oncotype DX, Prosigna, EndoPredict and MammaPrint for guiding adjuvant chemotherapy decisions in HR+ early breast cancer with 1–3 positive nodes, in terms of prognostic ability, prediction of chemotherapy benefit, impact on chemotherapy decisions, quality of life and anxiety. Searches covered MEDLINE, EMBASE and Cochrane databases in April 2023.

**Results:**

Fifty-five articles were included. All four tests were prognostic for distant recurrence in LN+ patients. The RxPONDER trial reported no chemotherapy benefit in post-menopausal LN+ patients with low Oncotype DX (RS 0–25), whilst pre-menopausal patients had statistically significant chemotherapy benefit. An RCT reanalysis of Oncotype DX (SWOG-8814) suggested greater chemotherapy benefit with higher RS in post-menopausal LN+ patients. The MINDACT trial reported that LN+ patients with high clinical risk and low MammaPrint risk had a non-statistically significant chemotherapy benefit, but was not designed assess differential chemotherapy benefit per risk group. Decisions to undergo chemotherapy reduced by 12–75% following Oncotype DX testing in LN+ patients in the UK and Europe. No studies in LN+ populations were identified for prediction of chemotherapy benefit by Prosigna or EndoPredict; or for chemotherapy decisions for Prosigna, EndoPredict or MammaPrint; or for anxiety or quality of life impact for any test.

**Conclusions:**

All four tests have prognostic ability in LN+ patients. Evidence on predictive benefit is weaker, with equivocal evidence that Oncotype DX may predict chemotherapy benefit in LN+ post-menopausal patients. Use of Oncotype DX leads to fewer patients being recommended chemotherapy.

**Supplementary Information:**

The online version contains supplementary material available at 10.1007/s10549-024-07596-0.

## Introduction

Many patients with hormone receptor-positive (HR+) lymph node-positive (LN+) early stage breast cancer (ESBC) receive adjuvant chemotherapy to reduce the risk of recurrence and improve survival [1]. However, chemotherapy often has considerable short- and long-term side effects. Improved information on recurrence risk and likely benefit of chemotherapy may help inform decisions about chemotherapy use for individual patients.

Currently, adjuvant chemotherapy decisions may be informed by clinical and pathological information, sometimes via a risk prediction tool. Various tools are used to estimate prognosis based on clinical and pathological factors, including age, tumour size, grade, nodal status, oestrogen receptor (ER) status, human epidermal growth factor receptor 2 (HER2) status, Ki67 status, menopausal status, comorbidities, frailty, mode of detection and generation of chemotherapy regimen. Prognostic tools include the Nottingham Prognostic Index, NPI [2], Adjuvant! Online, AOL [3], PREDICT [4] and, for older adults, the Age Gap Decision Tool [5].

Gene expression profiling (GEP) tests estimate an individual’s recurrence risk through integration of tumour biology and may also identify patients most likely to benefit from chemotherapy. This review covers four GEP tests: Oncotype DX, Prosigna, EndoPredict and MammaPrint (Box 1). All four tests measure the expression of cancer-related genes. Two tests (Prosigna and EndoPredict) incorporate clinical factors in the risk score. EndoPredict and MammaPrint each have two risk categories (high or low), whilst Prosigna has three risk categories (low, intermediate and high), and Oncotype DX previously had three risk categories whilst later publications use two.

Our group undertook a previous systematic review of GEP tests in both lymph node-negative (LN0) and LN+ ESBC [6] which informed the 2018 National Institute for Health and Care Excellence (NICE) Diagnostics Guidance 34, DG34 [7]. This guidance recommends Oncotype DX, Prosigna and EndoPredict for guiding chemotherapy decisions in ER+ HER2− LN0 ESBC including micrometastases; this recommendation is not restricted by menopausal status. Two other tests (MammaPrint and IHC4) were not recommended by NICE in LN0 populations. DG34 [7] also covered LN+ ESBC but did not make recommendations in this group due to insufficient data.

Meanwhile, the American Society of Clinical Oncology (ASCO) 2022 guideline update [8] recommends all four tests (Oncotype DX, Prosigna, EndoPredict and MammaPrint) in LN0 patients who are post-menopausal or aged > 50 years, but recommends only Oncotype DX in LN0 pre-menopausal patients. For LN+ disease, ASCO recommends Oncotype DX, EndoPredict and MammaPrint in post-menopausal or age > 50 populations, but does not recommend any tests in LN+ pre-menopausal patients.

This systematic review updates our previous review [6] to inform subsequent NICE guidance (DG58) [9] evaluating whether GEP tests (Oncotype DX, Prosigna, EndoPredict and MammaPrint) are clinically effective for guiding adjuvant chemotherapy decisions in HR+ HER2− ESBC with 1–3 positive nodes.

**Box 1:** Summary of gene expression profiling tests

Test	Oncotype DX Recurrence Score	Prosigna	EndoPredict EPclin score	MammaPrint
**Manufacturer**	Exact Sciences	Veracyte	Myriad	Agendia
**Description**	– 21 Gene assay (16 cancer genes) via RT-qPCR)	– 50 Gene assay (50 cancer genes) via direct mRNA counting – Also incorporates clinical factors	– 12 Gene assay (8 cancer genes) via RT-qPCR) – Also incorporates clinical factors	– 70 Gene assay (70 cancer genes) via microarray
**Outcomes assessed (according to manufacturer)**	– Distant recurrence risk – Chemotherapy benefit	– Distant recurrence risk – Intrinsic subtype	– Distant recurrence risk – Chemotherapy benefit	– Distant recurrence risk – Chemotherapy benefit
**Test result categories**^**a**^	Recurrence Score^a^ Original cut-offs: – Low: 0–17 – Intermediate: 18–30 – High: 31–100 RxPONDER cut-offs: – Low: 0–25 – High: 26–100	Risk category^a^ (Risk of Recurrence score, if 1–3 positive nodes) – Low: 0–15 – Intermediate: 16–40 – High: 41–100	Risk category^a^ – Low: < 3.3 – High: ≥ 3.3	Risk category^a^ – Low: Greater than 0 – High: 0 or less (– Ultra-low: greater than 0.355)
**Testing location**	Test service (USA)	Local laboratory	Local laboratory	Local laboratory (NGS) or test service (USA)
**Cancer stage**	Early stage (Stages I to IIIa)	Early stage (Stages I to IIIA)	Early stage	Early stage (Stages I, II or operable Stage III)
**Lymph node status**	LN0 or LN+ (up to 3 positive nodes)	LN0 or LN+ (up to 3 positive nodes and 4 + nodes)	LN0 or LN+ (up to 3 positive nodes)	LN0 or LN+ (up to 3 positive nodes)
**Hormone receptor status**	HR+	HR+	ER+	HR+
**HER2 status**	HER2−	HER2− or HER2+	HER2-	HER2−
**Menopausal status**	Pre- and post-menopausal	Post-menopausal only	Pre- and post-menopausal	Pre- and post-menopausal
**Treatment assumptions**	Score assumes 5 years of endocrine treatment	Score assumes 5 years of endocrine treatment	Scores assume 5 years of endocrine treatment	Scores assume 7 years of endocrine treatment^b^

*ER* oestrogen receptor, *HER2* human epidermal growth factor, *HR* hormone receptor, *LN* lymph node, *RT-qPCR* reverse transcription-quantitative polymerase chain reaction, *NGS* next generation sequencing, *mRNA* messenger ribonucleic acid, *USA* United States of America

^a^Risk cut-offs for Oncotype DX, EndoPredict and MammaPrint are the same for LN0 and LN+ populations, whilst Prosigna cut-offs differ for LN0 (low 0–40, intermediate 41–60, high 61–100), LN1–3 (low 0–15, intermediate 16–40, high 41–100) and LN4+ (high 0–100)

^b^In the MINDACT trial [10], 7 years of endocrine therapy was indicated for HR+ patients

## Methods

### Review question

This systematic review evaluates the effectiveness of four GEP tests (Oncotype DX, Prosigna, EndoPredict and MammaPrint), compared with current decision-making (no testing), to guide adjuvant chemotherapy decisions in people with HR+ HER2− ESBC with 1–3 positive nodes, in terms of prognostic ability, prediction of chemotherapy benefit, impact on chemotherapy decisions, and health-related quality of life (HRQoL) and anxiety associated with testing. A review protocol is available on PROSPERO (record CRD42023425638).

### Inclusion and exclusion criteria

#### Population

The relevant population was HR+, HER2−, ESBC with 1–3 positive lymph nodes (excluding micrometastases, which were included in NICE DG34 recommendations for LN0 patients). Studies were included if ≥ 80% of the population or subgroup were within scope; for example, if ≥ 80% were LN+ . However, to ensure inclusion of sufficient relevant evidence, studies not reporting HER2 status were included, as were studies in which ≥ 80% of subjects were LN+ but > 20% had > 3 positive nodes (limitations of such studies were noted).

#### Interventions

Relevant interventions (tests) included Oncotype DX, Prosigna, EndoPredict (EPclin score) and MammaPrint. Only studies using commercial versions of tests were included. The review excluded studies in which algorithms for genes within a test are applied to electronic (in silico) databases of genetic profiles generated from microarray techniques. The Prosigna risk of recurrence (ROR) score was included, as was the ROR-PT score which is equivalent to Prosigna (incorporates the PAM-50 gene signature, proliferation score and tumour size), but Prosigna intrinsic subtypes were excluded.

#### Comparators

The comparator for the review as a whole is current decision-making, including any tool or clinico-pathological features used to assess risk of recurrence. However, due to a lack of studies comparing GEP tests versus current tools, various evidence types were sought as outlined below, generally involving comparisons between test risk groups, or (for decision impact studies) comparisons pre and post testing.

#### Outcomes

The following outcomes were includable:

(i) Prognostic ability, i.e., the ability of a test to differentiate between patients with good versus poor outcomes, often expressed as a hazard ratio (HR) for risk of recurrence or mortality between risk groups;
(ii) Prediction of chemotherapy benefit, i.e., the ability to identify patients with differing relative benefit of chemotherapy, e.g. whether the HR for chemotherapy vs. no chemotherapy differs between test risk groups or ranges, generally assessed using statistical interaction tests [11];
(iii) Decision impact, i.e., the change in recommendations or decisions for use or non-use of chemotherapy before and after testing (restricted to UK and European studies due to differences in baseline chemotherapy use);
(iv) HRQoL and anxiety associated with testing.

For prognostic ability and prediction of chemotherapy benefit, relevant clinical outcomes included distant recurrence outcomes such as distant recurrence-free survival (DRFS), distant recurrence-free interval (DRFI), distant metastasis-free survival (DMFS), distant metastasis-free interval (DMFI) and distant recurrence-free rate (DRFR), as well as disease-free survival (DFS), invasive disease-free survival (IDFS), overall survival (OS) and breast cancer-specific survival (BCSS). Standardised endpoint definitions for adjuvant breast cancer trials have been reported previously [12, 13]. Local recurrence was not included.

#### Study types

Eligible data types included prospective randomised controlled trials (RCTs) of the tests, and study designs relevant to the above outcomes (described fully in “Results” section).

#### Date and language limits

No date limits were applied. Non-English studies were includable if sufficient data could be extracted; however, none were identified.

### Search strategy and study selection

Studies published before 2017 were identified from our previous review [6] and studies published from 2017 onwards via an updated search in April 2023. Searching covered databases, trial registers, conference proceedings, contact with experts, existing reviews and manufacturer submissions to NICE. Databases included MEDLINE, EMBASE, Cochrane, INAHTA and Web of Science (“Appendices A and B”). Search terms included test names and synonyms combined with terms for breast cancer (“Appendix A”). Titles and abstracts were assessed and 10% double checked early in the process to ensure consistency. Full-texts were assessed, and any uncertainties checked by a second reviewer.

### Data extraction and synthesis

Data were extracted into Microsoft Excel® and double checked. Data from studies published before 2017 were extracted directly from our previous review [6]. Results were presented via a narrative synthesis.

### Risk of bias assessment

Risk of bias in prospective RCTs was assessed using the Cochrane Risk of Bias tool Version 2 (RoB2) [14]. Prognostic and prediction studies were assessed using the Prediction model study Risk Of Bias Assessment Tool (PROBAST) [15]; items per domain were selected for relevance to this review, and definitions of risk per item defined a priori (“Appendix C”). Decision impact studies were discussed in terms of design and relevance but not formally quality assessed.

## Results

### Overview of evidence in LN+ populations

The search identified 4058 articles, of which 502 were checked as full-texts and 42 were includable (see PRISMA flow diagram, “Appendix B”). Thirteen additional articles were included from our previous review [6]. In total, 55 articles were included, 42 on prognostic and predictive ability and 13 on decision impact.

Evidence on prognostic ability in LN+ populations included the following study types. Firstly, reanalyses of clinical trials or cohorts, whereby tests are conducted on stored tumour samples from cohorts with long-term follow-up, allowed comparison of recurrence/survival outcomes between risk groups for all four tests. Secondly, two prospective RCTs provided prognostic data: RxPONDER [16] for Oncotype DX and MINDACT [10] for MammaPrint. In addition, the ongoing OPTIMA RCT compares Prosigna test-directed chemotherapy use vs. standard chemotherapy use, but results are not yet available. Thirdly, observational studies assessed the use of Oncotype DX in clinical practice.

Evidence assessing prediction of chemotherapy benefit in LN+ populations was identified for Oncotype DX and MammaPrint, but not for Prosigna or EPclin. For Oncotype DX, data included one trial reanalysis (SWOG-8814) [17] plus the RxPONDER RCT [16, 18]. For MammaPrint, data included a cohort reanalysis [19] and the MINDACT RCT [10].

Evidence assessing impact on chemotherapy decisions in LN+ populations in the UK and Europe was available from 12 studies of Oncotype DX, but not for other tests. No studies of anxiety or HRQoL impact associated with testing in a LN+ population were identified.

### Risk of bias

A summary of risk of bias in the included studies is provided here, with further details in “Appendix C”.

The two prospective RCTs (RxPONDER [16] and MINDACT [10]), assessed using the Cochrane RoB2 tool [14], scored low risk of bias on all domains and low risk of bias overall. However, there may have been selection bias in RxPONDER since patients had knowledge of their RS result before agreeing to be randomised.

Risk of bias in prognostic and predictive studies was assessed using the PROBAST tool [15]. For prognostic studies, the following factors may have affected results to some extent. Studies varied in terms of receipt of chemotherapy, and are therefore subgrouped by chemotherapy use. Some participants did not match the review question (either not all HR+, not all HER2− or not all LN1–3). Most studies excluded some patients for reasons including insufficient tissue, missing data, failed tests and others, though the potential impact on results is unclear. Randomisation to chemotherapy or no chemotherapy only occurred in the RxPONDER and MINDACT prospective RCTs and in the SWOG-8814 [17] RCT reanalysis, whilst in observational studies, chemotherapy use was not randomised. Allocation to chemotherapy or no chemotherapy was not influenced by the test result in studies using retrospective testing (i.e., reanalyses of RCTs and cohorts), whereas in observational studies with prospective use of testing, test results may have influenced chemotherapy use.

### Trial and cohort reanalyses: prognostic ability for all tests

A summary of prognostic data in LN+ populations for 10-year distant recurrence across all four tests, based on reanalyses of trials or cohorts, is provided in Table 1 (with full details in “Appendix D”). Most studies enrolled post-menopausal populations. Results are grouped into studies of endocrine monotherapy, and studies using chemotherapy in some or all patients. All trial reanalyses assessing Oncotype DX used the older cut-offs of RS 18 and 30; none used the RS ≤ 25 cut-off.

**Table 1:** Summary of prognostic data for 10-year distant recurrence (all four tests)

Test	ET/CT	Reference study	Design	*N* pts	Outcome	Nodal status	HR, HER2	Meno status	Test cut-offs	Distribution %			DR free 0–10 yr %			10 yr HR sig?^a^	
										Low	Int	High	Low	Int	High	Unadj	Adj
Oncotype DX	ET alone	Sestak [20, 37] (TransATAC)	RCT-R	183	DRFI	LN1–3	HR+ HER2−	Post	18, 30	57	32	11	81	71	62	N	N
	All CT + ET	Mamounas [21] (NSABP-28)	RCT-R	722	DRFI	LN1–3	ER+ NR HER2	Pre/post	18, 30	37	34	28	85	72	63	Y	Y
MammaPrint	Variable ET/CT	Drukker [22]	Cohort-R	144	DMFS	74% LN1–3 26% LN4+	77% ER+ NR HER2	Pre/post (age < 53)	0.4	38	–	62	79	–	54	Y	–
		Mook [19]	Cohort-R	241	DMFS	LN1–3+ LNmicro	79% ER+ 84% HER2−	Pre/post	NR	41	–	59	91	–	76	Y	N
		Vliek [23] (RASTER)	Cohort-R	134	DRFI	LN1–3	83% ER+ 85% HER	Pre/post	NR	48	–	52	95	–	81	Y	–
Prosigna	ET alone	Sestak [20, 37] (TransATAC)	RCT-R	183	DRFI	LN1–3	HR+ HER2 −	Post	16, 40	8	32	60	100	79	69	N	Y
		Gnant [24]/Filipits [25] (ABCSG-8)	RCT-R	413	DMFS	89% LN1–3 11% LN4+	ER+ HER2−	Post	16, 40	4	34	62	100	94	76	–	Y
		Laenkholm [26] **(**DBCG)	Cohort-R	1395	DRFS	LN1–3	HR+ HER2−	Post	Varies by *N* nodes^b^	26	28	46	97	89	78	Y	Y
	All CT + ET	Martin [27] (GEICAM 9906)	RCT-R	536	DMFS	64% LN1–3 36% LN4+	ER+ HER2−	54% Pre 46% Post	18, 65	19	56	26	92	74^c^	66^c^	Y	N
EndoPredict (EPclin)	ET alone	Sestak [20, 37] (TransATAC)	RCT-R	183	DRFI	LN1–3	HR+ HER2−	Post	3.3	23	–	77	94	–	70	Y	Y
		Filipits [29] (ABCSG-6/8)	RCT-R	453	DRFR	LN1–3	ER+ HER2−	Post	3.3	35	–	65	96	–	81	Y	Y
		Constantinidou [30]	Cohort-R	62	DRFS	LN1–3	ER+ HER2−	Pre	3.3	19	–	81	100	–	75	N	Y
	All CT + ET	Martin [27, 28] (GEICAM 9906)	RCT-R	555	DMFS	64% LN1–3 36% LN4+	ER+ HER2−	54% Pre 46% Post	3.3	13	–	87	100	–	72	Y	Y

*Hyphen* not reported, *Adj* adjusted, *cohort-R* cohort reanalysis, *CT* chemotherapy, *DMFS* distant metastasis-free survival, *DR* distant recurrence, *DRFI* distant recurrence-free interval, *DRFR* distant recurrence-free rate, *DRFS* distant recurrence-free survival, *ET* endocrine therapy, *HER2* human epidermal growth factor receptor 2, *HR* hazard ratio, *HR* hormone receptor, *int* intermediate, *LN* lymph nodes (number positive), *meno* menopausal, *NR* not reported, *prog* prognostic, *RCT* randomised controlled trial, *RCT-R* RCT reanalysis, *sig* significant, *unadj* unadjusted, *var* variable, *yr* year

^a^The last two columns indicate how many studies report an HR between test risk groups which is statistically significant at the 5% level (unadjusted or adjusted for clinical factors)

^b^Laenkholm 2018 cut-offs: 1 positive node: low ≤ 35, intermediate 36–55, high > 55; 2 positive nodes: low ≤ 25, intermediate 26–45, high > 45; 3 positive nodes: none low, intermediate ≤ 25, high > 25

^c^Martin 2016 (Prosigna): data extracted for ROR-PT score (equivalent to Prosigna); 10-year DMFS for intermediate and high groups estimated from the plot in Fig. 1 of the paper

Firstly, these studies provide data on the proportion of patients classed as low-, intermediate- or high-risk by each test. More patients were assigned to the low-risk group by Oncotype DX [20, 21] (37–57% low-risk) and MammaPrint [19, 22, 23] (38–48% low-risk) than by Prosigna [20, 24–28] (4–26% low-risk) or EPclin [20, 27–30] (13–35% low-risk). This has implications regarding how many patients may receive chemotherapy in practice following use of the different tests [31].

Secondly, these studies report the proportion of patients experiencing distant recurrence per test risk group. In the low-risk groups (amongst studies of endocrine monotherapy), freedom from distant recurrence at 10 years was 81% (Oncotype DX [20]), 100% (Prosigna [20, 24], excluding the study [26] using non-standard cut-offs) and 94–100% (EPclin [20, 29, 30]). As may be expected for prognostic tests, more patients experienced recurrence in the high-risk groups, with freedom from distant recurrence at 10 years of 62% (Oncotype DX [20]), 69–76% (Prosigna [20, 24]) and 70–81% (EPclin [20, 29, 30]). Studies in which some or all patients received chemotherapy showed a similar pattern (Table 1). For MammaPrint, no studies involved endocrine monotherapy, whilst in studies with some use of chemotherapy, freedom from distant recurrence at 10 years was 79–95% (low-risk) and 54–81% (high-risk) [19, 22, 23].

Thirdly, these studies can assess whether tests were significantly prognostic for 10-year distant recurrence in LN+ populations. Across all four tests, there were statistically significant differences in outcomes between test risk groups within many (though not all) analyses, both with and without adjustment for clinical factors (see last two columns of Table 1; full results including HRs between risk groups are presented in “Appendix D”).

Some of the above studies, plus additional studies [17, 32–36], reported prognostic ability for other outcomes such as DFS, OS and BCSS, or reported distant recurrence at 5 years rather than 10 years (“Appendix D”). Again, analyses of these outcomes suggest statistically significant prognostic ability for all four tests on many (though not all) analyses.

### Trial reanalysis: prediction of chemotherapy benefit for Oncotype DX

Reanalyses of trials of chemotherapy vs. no chemotherapy can assess whether a test is predictive for chemotherapy benefit. In a reanalysis of the SWOG-8814 RCT [17], Oncotype DX was conducted retrospectively on tumour samples from LN+ post-menopausal patients randomised to chemotherapy vs. no chemotherapy (Table 2). This RCT did not report distant recurrence. For 10-year DFS, using cut-offs of RS 18 and 30, adjusted HRs indicated no effect of chemotherapy in the low-risk group [RS 0–17: HR 1.02; 95% confidence interval (CI) 0.54 to 1.93]; and a non-statistically significant effect in the intermediate-risk group (RS 18–30: HR 0.72; 95% CI 0.39 to 1.31); whilst the effect of chemotherapy in the high-risk group was not statistically significant but the upper 95% CI limit was close to excluding 1.0 (RS > 30: HR 0.59; 95% CI 0.35 to 1.01). Interaction tests between chemotherapy effect and linear recurrence score (for 10-year DFS) were statistically significant when adjusted for various clinical factors (*p*-value not reported), but non-significant when adjusting for number of positive nodes (*p* = 0.53) or Allred-scored ER status (*p* = 0.15). Interaction tests for DFS were significant for the period 0–5 years but not for the period 5–20 years. Results for 10-year BCSS and OS were similar, with significant or borderline significant effects of chemotherapy in the high-risk group only, and some significant interaction tests (Table 2). In summary, this study suggested that patients with higher Oncotype DX scores may have a greater relative benefit from chemotherapy, but this was not conclusive.

**Table 2:** Prediction of chemotherapy benefit: RCT and cohort reanalysis (Oncotype DX and MammaPrint)

Study Reference Design	Nodal status HR, HER2 (*N*)	Outcome	Test cut-offs	% Risk of outcome						Prediction of chemotherapy benefit								
				Low		Int		High		Absolute difference (CT vs. no CT)			HR for CT vs. no CT (95% CI), *p*-value^a^				Interaction RS and CT	Sig pred?^b^
				CT	No	CT	No	CT	No	Low	Int	High	Low	Int	High	Adj		
Oncotype DX																		
SWOG-8814 Albain [17] RCT-R	Post-meno LN1–3: 62% LN4+: 38% 100% HR+ 88% HER2− (*n* = 367)	DFS 0–10 yr	18, 30	64	60	–	–	55	43	4%	–	12%	1.02 (0.54 to 1.93) SLR *p* = 0.97	0.72 (0.39 to 1.31) SLR *p* = 0.48	0.59 (0.35 to 1.01) SLR *p* = 0.033	Y	0–10 yr: *p* = 0.053 (adj nodes) *p* = sig (NR) (adj various) *p* = 0.15 (adj Allred-ER)	N Y N
		DFS 0–5 yr	18, 30	–	–	–	–	–	–	–	–	–	1.34 (0.47 to 3.82)	0.95 (0.43 to 2.14)	0.59 (0.32 to 1.11)	Y	0–5 yr: *p* = 0.029 (adj nodes)	Y
		DFS 5–10 yr	18, 30	–	–	–	–	–	–	–	–	–	0.88 (0.38 to 1.92)	0.52 (0.21 to 1.27)	0.60 (0.22 to 1.62)	Y	5–10 yr: *p* = 0.58 (cont RS, adj nodes)	N
		BCSS 0–10 yr	18, 30	–	–	–	–	73	54	–	–	19%	SLR *p* = 0.56	SLR *p* = 0.89	SLR *p* = 0.033	Y	–	–
		OS 0–10 yr	18, 30	–	–	–	–	68	51	–	–	17%	1.18 (0.55 to 2.54); *p* = 0.68 SLR *p* = 0.63	0.84 (0.40 to 1.78); *p* = 0.65 SLR *p* = 0.85	0.56 (0.31 to 1.02); *p* = 0.057 SLR *p* = 0.027	Y	0–10 yr: *p* = 0.026 (adj nodes) 0–5 yr: *p* = 0.016 (adj nodes) 5–10 yr: *p* = 0.87 (adj nodes)	Y Y N
MammaPrint																		
Two cohorts Mook [19] Cohort-R	All ages LN1micro to LN3 79% ER+ 84% HER2− (*n* = 347)	BCSS 0–10 yr	NR	–	–	N/A	N/A	–	–	–	N/A	–	–	N/A	–	–	0–10 yr:* p* = 0.95 (adj)	N

*Hyphen* not reported, *Abs diff* absolute difference, *adj* adjusted, *BCSS* breast cancer-specific survival, *CI* confidence interval, *cohort-R* cohort reanalysis, *CT* chemotherapy, *DFS* disease-free survival, *ER* oestrogen receptor, *HER2* human epidermal growth factor receptor 2, *HR* hazard ratio, *HR* hormone receptor, *int* intermediate, *LN* lymph nodes (number positive), *meno* menopausal, *NR* not reported, *OS* overall survival, *prosp* prospective, *pred* predictive of CT benefit, *RCT* randomised controlled trial, *RCT-R* RCT reanalysis, *RS* – Recurrence Score (Oncotype DX), *sig* significant, *SLR* stratified log-rank, *unadj* unadjusted, *yr* year

^a^Stratified log-rank (SLR) *p*-values reported in Albain et al. [17] do not always match

^b^Indicates whether statistically significant for prediction of chemotherapy benefit (i.e. whether interaction test between linear test score and effect of CT via Cox model is significant at 5% level)

### Cohort reanalysis: prediction of chemotherapy benefit for MammaPrint

A publication from 2009 [19] reporting a reanalysis of two cohorts in LN+ populations (*N* = 347) reported a non-significant interaction test between MammaPrint score and effect of chemotherapy on 10-year BCSS (*p* = 0.95), therefore did not provide evidence for the ability of MammaPrint to predict different relative benefits of chemotherapy (Table 2).

### Prospective RCT (RxPONDER): prognostic ability for Oncotype DX

RxPONDER (Table 3) is a prospective RCT [16, 18, 38] of patients with HR+ HER2− LN+ ESBC with lower Oncotype DX scores (RS 0–25). Participants were randomised to chemotherapy versus no chemotherapy, with all patients receiving endocrine therapy. Patients with RS > 25 were not included. The data in Table 3 are based on the main RxPONDER publication [16] for the full trial population, and based on a subsequent conference presentation [18] for the pre- and post-menopausal populations. This is because the conference presentation includes data on DRFI, and has slightly longer follow-up (median 6.1 years versus 5.3 years), but only reports data according to menopausal status. There were no major differences between the two sources.

**Table 3:** RxPONDER RCT of Oncotype DX: prognostic and predictive ability

Outcome	*N*	Test cut-offs	% Risk of outcome		Prognostic ability		Prediction of chemotherapy benefit			
					HR per unit RS change (95% CI) within RS 0–25	Sig prog?^a^	Absolute diff CT vs. no CT	HR for CT vs. no CT (95% CI) within RS 0–25	Interaction RS and CT	Sig pred?^b^
			CT	No CT						
**Full population (LN1–3, HR+, HER2−)** [16, 18]										
DRFS (0–5 yr)	*n* = 4984	RS ≤ 25	94.9	93.9	–	–	1.0	HR 0.88 (0.71 to 1.09), *p* = 0.25	–	–
IDFS (0–5 yr)	*n* = 4984	RS ≤ 25	92.2	91.0	HR per unit RS (adj meno and CT): 1.05 (1.04 to 1.07), *p* < 0.001	Y	1.2	HR 0.86 (0.72 to 1.03), *p* = 0.10	HR 1.02 (0.98 to 1.05), *p* = 0.35 (adj meno)	N
**Post-menopausal**										
DRFI^c^ (0–5 yr)	*n* = 3329	RS ≤ 25	95.8	96.6	–	–	− 0.8	Adj HR 1.12 (0.82 to 1.52), *p* = 0.49	–	–
DRFS^c^ (0–5 yr)	*n* = 3329	RS ≤ 25	94.3	94.8	–	–	− 0.5	Adj HR 1.12 (0.88 to 1.44), *p* = 0.35	–	–
IDFS^c^ (0–5 yr)	*n* = 3329	RS ≤ 25	91.2	91.9	HR per unit RS (adj for CT, nodes, grade, tumour size, age): 1.05 (1.03 to 1.07), *p* < 0.001	Y	− 0.7	Adj HR 1.06 (0.87 to 1.30), *p* = 0.55	HR 1.01 (0.97 to 1.06), *p* = 0.48	N
**Pre-menopausal**										
DRFI^c^ (0–5 yr)	*n* = 1655	RS ≤ 25	96.3	93.9	–	–	2.4	Adj HR 0.64 (0.43 to 0.95), *p* = 0.026	–	–
DRFS^c^ (0–5 yr)	*n* = 1655	RS ≤ 25	95.9	93.4	–	–	2.5	Adj HR 0.66 (0.45 to 0.97), *p* = 0.033	–	–
IDFS^c^ (0–5 yr)	*n* = 1655	RS ≤ 25	93.9	89.0	HR per unit RS (adj for CT, nodes, grade, tumour size, age): 1.06 (1.02 to 1.09), *p* = 0.001	Y*	4.9	Adj HR 0.64 (0.47 to 0.87), *p* = 0.004	HR 1.04 (0.97 to 1.12), *p* = 0.26	N

*Abs diff* absolute difference, *adj* adjusted, *CI* confidence interval, *CT* chemotherapy, *DRFI* distant recurrence-free interval, *DRFS* distant recurrence-free survival, *HER2* human epidermal growth factor receptor 2, *HR* hazard ratio, *HR*+ hormone receptor positive, *IDFS* invasive disease-free survival, *LN* lymph nodes (number positive), *meno* menopausal status, *NR* not reported, *prosp* prospective, *pred* predictive of CT benefit, *RCT* randomised controlled trial, *RS* Recurrence Score (Oncotype DX), *sig* significant, *unadj* unadjusted, *yr* year, *Hyphen* not reported

^a^Indicates whether statistically significant for prognostic ability (i.e. whether HR per unit change in RS is significant at the 5% level)

^b^Indicates whether statistically significant for prediction of chemotherapy benefit (i.e. whether interaction test between RS and effect of CT is significant at the 5% level)

^c^Additional RxPONDER data from Kalinsky et al. (2022) SABCS slides [18] (in addition to main publication, Kalinsky et al. [16])

Freedom from distant recurrence at 5 years (DRFS and DRFI) ranged from 93 to 97% for the full study population (RS 0–25), as well as for pre-menopausal and post-menopausal subgroups, both with and without chemotherapy. For comparison, in two RCT reanalyses [20, 32], 5-year DRFI was 96% and 94% respectively in the RS 0–17 group, and 85% and 87% respectively in the RS 18–30 group.

In RxPONDER, no statistical analyses of prognostic ability were reported for distant recurrence. However, Oncotype DX as a continuous score was statistically significantly prognostic for 5-year IDFS within the study population of RS 0–25, after adjusting for clinical factors (HR per unit change in RS was 1.05; 95% CI 1.04 to 1.07; *p* < 0.001), with similar significant results in the pre-menopausal and post-menopausal subgroups (Table 3).

### Prospective RCT (RxPONDER): prediction of chemotherapy benefit for Oncotype DX

The RxPONDER RCT [16, 18] (Table 3) also aimed to evaluate whether chemotherapy could be avoided in LN+ populations with lower genomic risk (RS 0–25). RxPONDER demonstrated no benefit of adjuvant chemotherapy in LN+ post-menopausal patients with RS 0–25 (5-year DRFI was 95.8% with chemotherapy vs. 96.6% with no chemotherapy, an absolute difference of 0.8% favouring no chemotherapy; adjusted HR 1.12; 95% CI 0.82 to 1.52; *p* = 0.49). Conversely, there was a statistically significant chemotherapy benefit in pre-menopausal patients with RS 0–25 (5-year DRFI was 96.3% with chemotherapy vs. 93.9% with no chemotherapy, an absolute difference of 2.4% favouring chemotherapy; adjusted HR 0.64; 95% CI 0.43 to 0.95; *p* = 0.026).

RxPONDER was not designed to assess the relationship between RS and effect of chemotherapy for RS > 25, though this relationship could be assessed within the RS range 0–25. No interaction test was reported for distant recurrence. For IDFS, a test for interaction between RS (within the range 0–25) and chemotherapy effect was not statistically significant, either across all patients (HR 1.02; 95% 0.98 to 1.05; *p* = 0.35) or in the pre-menopausal or post-menopausal subgroups (Table 3), indicating no significant predictive ability of Oncotype DX for chemotherapy benefit within the range RS 0–25.

### Prospective RCT (MINDACT): prognostic ability for MammaPrint

The MINDACT RCT [10] (Table 4) assessed patients’ genomic risk via MammaPrint, and clinical risk via modified AOL (mAOL). Patients who were low-risk on both measures were allocated to no chemotherapy, those who were high-risk on both were allocated to chemotherapy, and patients with discordant risk were randomised to chemotherapy vs. no chemotherapy. Data for LN+ patients could only be analysed within the clinical high-risk subgroup (since in the clinical low-risk subgroup, LN+ patients with MammaPrint high-risk were not reported due to low numbers).

**Table 4:** MINDACT RCT of MammaPrint: prognostic and predictive ability

Outcome	*N*	Clinical risk^c^	Age	Test cut-offs	% Risk of outcome				Prognostic ability	Prediction of chemotherapy benefit				
					Low MMP		High MMP		HR between risk groups	Abs diff CT vs. no CT		HR for CT vs. no CT (95% CI)		Interaction RS and CT
					CT	No CT	CT	No CT		Low MMP	High MMP	Low MMP	High MMP	
**All LN1–3 population (HR+, HER2−)** [10]														
DMFS^a^ (0–8 yr)	658	High mAOL	All ages	> 0 Low, ≤ 0 high	91.2	89.9	79.1	–	–	1.3	–	HR 0.84 (0.51 to 1.37), *p* = NR	–	–
DMFI^a^ (0–8 yr)	658	High mAOL	All ages	> 0 Low, ≤ 0 high	92.3	90.9	80.9	–	–	1.4	–	HR 0.85 (0.50 to 1.44), *p* = NR	–	–
DFS^a^ (0–8 yr)	658	High mAOL	All ages	> 0 Low, ≤ 0 high	85.3	82.8	74.5	–	–	2.5	–	–	–	–
OS^a^ (0–8 yr)	658	High mAOL	All ages	> 0 Low, ≤ 0 high	95.5	94.9	89.1	–	–	0.6	–	–	–	–
**Older age group (> 50 years, LN1–3, HR+, HER2−)**														
DMFI^b^ (0–8 yr)	NR	High mAOL	> 50 yr	> 0 Low, ≤ 0 high	91.4	91.2	–	–	–	0.2	–	Adj HR 0.88 (0.46 to 1.68), *p* = NR	–	–
OS^b^ (0–8 yr)	NR	High mAOL	> 50 yr	> 0 Low, ≤ 0 high	94.8	95.9	–	–	–	-1.1	–	Adj HR 0.99 (0.45 to 2.18), *p* = NR	–	–

*Abs diff* absolute difference, *adj* adjusted, *CI* confidence interval, *CT* chemotherapy, *DFS* disease-free survival, *DMFI* distant metastasis-free interval, *DMFS* distant metastasis-free survival, *HER2* human epidermal growth factor receptor 2, *HR* hazard ratio, *HR*+ hormone receptor positive, *LN* lymph nodes (number positive), *mAOL* modified Adjuvant! Online, *meno* menopausal, *NR* not reported, *OS* overall survival, *prosp* prospective, *pred* predictive of CT benefit, *RCT* randomised controlled trial, *sig* significant, *unadj* unadjusted, *yr* year, *Hyphen* = not reported

^a^Data from the Piccart et al. supplement ([10], Tables S10 and S12)

^b^Data provided by Agendia during NICE technology assessment; available at https://www.nice.org.uk/guidance/dg58. Not reported which factors the HRs are adjusted for

^c^All data are from the mAOL high-risk subgroup. Data for the mAOL low-risk, MammaPrint high-risk group were not reported in the publication due to small numbers of LN+ patients (*n* = 15)

Within LN+ clinical high-risk patients, freedom from distant recurrence at 8 years (DMFI) in patients receiving chemotherapy was more favourable in the MammaPrint low-risk group (92.3%) than the MammaPrint high-risk group (80.9%), suggesting a prognostic effect of the test. Results for 8-year DMFS were similar (Table 4). A further publication reported 8-year DMFI of 95.2% in ultra-low-risk patients (MammaPrint score > 0.355) [39]. However, no HRs or significance tests were reported between MammaPrint risk groups, so prognostic ability could not be formally determined.

### Prospective RCT (MINDACT): prediction of chemotherapy benefit for MammaPrint

Within the LN+ , clinical high-risk, MammaPrint low-risk group of MINDACT [10], 8-year DMFS was 91.2% with chemotherapy vs. 89.9% with no chemotherapy, an absolute difference of 1.3% favouring chemotherapy, with a non-significant HR (HR 0.84; 95% CI 0.51 to 1.37; Table 4). In the same subgroup but restricted to older patients (age > 50 years), 8-year DMFI was 91.4% with chemotherapy vs. 91.2% with no chemotherapy, an absolute difference of 0.2%, with a non-significant HR (adjusted HR 0.88; 95% CI 0.46 to 1.68). No data were reported for LN+ patients aged ≤ 50 years. The effect of chemotherapy could not be determined in the clinical high-risk, MammaPrint high-risk group, since all such patients were offered chemotherapy. Without this comparison, it was not possible to determine from MINDACT whether MammaPrint was predictive for chemotherapy benefit.

### Observational and registry data: prospective use of Oncotype DX

Observational and registry studies reported prospective use of Oncotype DX in LN+ patients in clinical practice. These studies provide large-sample real-world data, but are limited because test results likely influenced chemotherapy use and therefore outcomes. These studies included the US National Cancer Database, NCDB [40–44] (*n* = 25,029), the US Surveillance Epidemiology and End Results (SEER) registry [45–47] (*n* = 6483), the Clalit registry [48] in Israel (*n* = 709) and a few smaller prospective studies [49–51]. Data on distant recurrence are shown in Table 5, and other outcomes in “Appendix E”.

**Table 5:** Observational and registry data for Oncotype DX (distant recurrence)

Cohort	Outcome	*N*	Age	Nodal status HR, HER2 ET/CT	Test cut-offs	Distribution %				% Risk of outcome						Prognostic ability							
						Low	Int	High	Low			Int		High		HR between test risk groups (95% CI)							Sig prog?^a^
**Prognostic ability: All ages (distant recurrence)**																							
Clalit, Israel [48]	DRFI (0–5 yr)	709	All ages	LN1micro: 42% LN1–3: 58% 100% ER+ 100% HER2− Var ET/CT	18, 30	53	36	10	97 (7% CT)			94 (40% CT)		83 (86% CT)		Low vs. high: HR 0.19 (0.09 to 0.40) Int vs. high: HR 0.39 (0.20 to 0.79), *p* < 0.001 Adj HR: Low vs. high: HR 0.23 (0.11 to 0.50) Adj HR: Int vs. high: HR 0.42 (0.20 to 0.86), *p* = 0.001							Y Y Y Y
					25	81		19	96 (15% CT)					87 (77% CT)		*p* < 0.001							Y
**Prognostic ability: Older age groups (distant recurrence)**																							
Clalit, Israel [48]	DRFI (0–5 yr)	464	Age 50–69	See above	18, 30	54	37	9	98 (6% CT)			94 (42% CT)		88 (90% CT)		*p* = 0.017							Y
	DRFI (0–5 yr)	136	Age ≥ 70	See above	18, 30	57	33	10	95 (7% CT)			89 (22% CT)		93 (57% CT)		*p* = 0.458							N
**Prognostic ability: Younger age groups (distant recurrence)**																							
Clalit, Israel [48]	DRFI (0–5 yr)	109	Age < 50	See above	18, 30	48	37	16	96 (12% CT)			100 (48% CT)		64 (100% CT)		*p* < 0.001							Y
YWBCS [49]	DRFS (0–6 yr)	163	Age ≤ 40	LNmicro, LN1–3 100% ER+ 100% HER2− Var ET/CT	18, 30	33	42	25	86 (83% CT)			87 (97% CT)		63 (98% CT)		*p* = 0.004							Y
					11, 25	9	54	37	92 (79% CT)			85 (92% CT)		71 (97% CT)		*p* = 0.10							N

Cohort	Outcome	*N*	Age	Nodal status HR, HER2 ET/CT (*N*)	Test cut-offs	Distribution %			% Risk of outcome							Prediction of chemotherapy benefit							
						Low	Int	High	Low			Int		High		Abs diff CT vs. no CT			HR: CT vs. no CT (95% CI)			Interaction	Sig pred^b^
									CT		No	CT	No	CT	No	Low	Int	High	Low	Int	High		
**Prediction of chemotherapy benefit: All ages (distant recurrence)**																							
Clalit, Israel [48]	DRFI 0–5 yr	709	All ages	See above	18, 30	–	–	–	92.3		97.1	99	90.3	82	90	− 4.8	8.7	− 8.0	*p* = 0.245	*p* = 0.019	–	–	–
					25	–	–	–	97.7		95.6	–	–	97.5	79.7	2.1		17.8	*p* = 0.521		*p* = 0.017	–	–
**Prediction of chemotherapy benefit: Older age groups (OS)**																							
NCDB [43] (Ductal)	OS 0–5 yr	NR	Age 50–75	LN1–3 100% HR+ 100% HER2−	≤ 25	–	–	–	–		–	–	–	–	–	–		–	Adj HR: 1.12 (0.86 to 1.46)		–	–	–
NCDB [44]	OS 0–5 yr	NR	Age 51–70	See above	≤ 25	–	–	–	–		–	–	–	–	–	1.6		–	Adj HR: 1.49 (1.12 to 1.97), *p* = 0.006		–	–	–
			Age > 70		≤ 25	–	–	–	–		–	–	–	–	–	–		–	Adj HR: 1.1 (0.68 to 1.78), *p* = 0.69		–	–	–

*Abs diff* absolute difference, *adj* adjusted, *CI* confidence interval, *CT* chemotherapy, *DRFI* distant recurrence-free interval, *DRFS* distant recurrence-free survival, *ER* oestrogen receptor, *ET* endocrine therapy, *HER2* human epidermal growth factor receptor 2, *HR* hazard ratio, *HR* hormone receptor, *int* intermediate, *LN* lymph nodes (number positive), *NR* not reported, *prosp* prospective, *pred* predictive of CT benefit, *RS* Recurrence Score (Oncotype DX), *sig* significant, *var* variable, *YWBCS* Young Women’s Breast Cancer Study, *yr* year, *Hyphen* not reported

^a^Indicates whether statistically significant for prognostic ability (i.e. whether HR between test risk groups is statistically significant at the 5% level)

^b^Indicates whether statistically significant for prediction of chemotherapy benefit (i.e. whether interaction test between RS and effect of CT is significant at the 5% level)

Regarding the proportion of patients allocated to each risk group, using cut-offs of RS 18 and 30, based on the Clalit [48] and SEER [45, 46] registries, 53–58% were low-risk (RS 0–17), 35–36% intermediate-risk (RS 18–30) and 7–10% high-risk (RS ≥ 30), which is similar to the distribution in the TransATAC study [20] (57% low, 32% intermediate, 11% high). Using an RS cut-off of 25, across the Clalit [48] and NCDB [40, 41] registries, the distribution ranged from 81 to 88% low-risk (RS 0–25) and 13–19% high-risk (RS > 25).

Distant recurrence outcomes per risk group were reported in two sources: Clalit [48] and the Young Women’s Breast Cancer Study, YWBCS [49] (Table 5). Within Clalit [48], using RS cut-offs of 18 and 30, Oncotype DX was significantly prognostic for freedom from distant recurrence at 5 years (97% for low-risk, 94% intermediate-risk, 83% high-risk; *p* ≤ 0.001) despite higher chemotherapy use in higher-risk groups. Oncotype DX was also significantly prognostic within Clalit using the newer RS cut-offs (with 5-year DRFI of 96% for RS ≤ 25 and 87% for RS > 25, *p* < 0.001).

In analyses of distant recurrence by age group, Oncotype DX was significantly prognostic in younger patients (age < 50 and ≤ 40 years, respectively) in Clalit [48] and YWBCS [49]. In older patients in Clalit, Oncotype DX was significantly prognostic in those aged 50–69 years, but not in those aged ≥ 70 years, though the latter group had smaller patient numbers (Table 5).

Data on other outcomes are shown in “Appendix E”. For BCSS and OS, most analyses of the Clalit [48], SEER [45, 46] and NCDB [40, 41, 44] registries showed a prognostic effect of Oncotype DX using cut-offs of either RS 18 and 30, or RS 11 and 25. Subgroup analyses of SEER reported statistically significant prognostic ability in white patients but non-significant results in black or other ethnicities (though these analyses were based on small numbers) [45], whilst statistically significant prognostic ability was reported in both men and women [47].

### Observational and registry data: prediction of chemotherapy benefit for Oncotype DX

Studies based on the Clalit [48], SEER [45–47] and NCDB [40–44] registries also reported outcomes per risk group for LN+ patients with and without chemotherapy, with the limitation that the use or non-use of chemotherapy was not randomised.

The Clalit registry [48, 52] was the only study to report 5-year distant recurrence data (Table 5). Using the cut-offs RS 18 and 30, the relationship between risk group and effect of chemotherapy was unclear (results favoured chemotherapy in the intermediate-risk group, but favoured no chemotherapy in the low- and high-risk groups). Using the cut-off RS ≤ 25 suggested a greater effect of chemotherapy in the higher-risk group (no significant chemotherapy benefit for RS ≤ 25; significant benefit for RS > 25); however, no formal interaction tests were reported. Data from Clalit [48], SEER [45–47, 53] and NCDB [40–44, 54–57] on other outcomes (such as BCSS and OS) are shown in “Appendix F”. No interaction tests were reported, and there was no clear pattern for chemotherapy effect in different RS ranges.

Since a key finding of RxPONDER was a lack of chemotherapy benefit in post-menopausal patients with RS 0–25, results from registry studies for older-age subgroups were sought. No data on distant recurrence were identified; however, the NCDB database reported 5-year OS within older-age subgroups (Table 5). Some analyses showed a significant effect of chemotherapy for RS ≤ 25 whilst others did not; therefore, the results did not clearly either support or refute the RxPONDER findings.

### Decision impact studies for Oncotype DX in LN+ populations (UK and Europe)

Decision impact studies, which assess changes in recommendations or decisions on whether to use chemotherapy before and after testing, were identified from the UK and Europe. Twelve studies of Oncotype DX in LN+ populations were identified (UK = 5, Italy = 4, Spain = 2, Germany = 1) [58–70]. No decision impact studies in LN+ populations were identified for EndoPredict, Prosigna or MammaPrint. A summary is provided in Table 6 (with full results in “Appendix G”).

**Table 6:** Decision impact: Oncotype DX

Reference, years Country (years)	Nodal status Clinical risk	Recommendation/decision	Menopausal status	*N* pts	Pre-test CT	Net change in CT decision/recommendation					
						Overall	RS 0–17	RS 18–30	RS 31–100	RS 0–25	RS 26–100
Holt 2024 [59, 70] **UK** (2017–2022)	LN1–3	R–D	All	664	530 (80%)	− 342 (− 52%)	− 271 (− 68%)	− 72 (− 35%)	+ 1 (+ 1.7%)	− 347 (− 61%)	+ 5 (+ 5%)
			Pre-meno	152	123 (81%)	− 52 (− 34%)				− 53 (− 42%)	+ 1 (+ 4%)
			Post-meno	512	407 (79%)	− 290 (− 57%)				− 294 (− 67%)	+ 4 (+ 5%)
Dieci 2019 [65] **Italy** (2017–2018)	LN1–3; 94% high clinical risk	R–R	All	99	54 (55%)	− 27 (− 27%)				− 27 (− 29%)	No change
Zambelli 2020 [68] **Italy** (2017–2018)	LN1–3; intermediate clinical risk	R–R	All	127	48 (38%)	− 23 (− 18%)	− 14 (− 20%)	− 9 (− 19%)	No change		
Llombart-Cussac 2023 [67] **Spain** (2016–2017)	LN1–3; high clinical risk; CT indicated	R–R	All	150	150 (100%)	− 109 (− 73%)	− 78 (− 91%)	− 31 (− 54%)	No change		
Loncaster 2017 [60] **UK** (2012–2015)	LN+ ; CT indicated; post-test decision based on RS	R–D	Post-meno	65	65 (100%)	− 45 (− 69%)	− 37 (− 93%)	− 7 (− 37%)	− 1 (− 17%)		
Nanda 2021 (abst) [62] **UK** (2013–2019)	LN1–3 (incl. micromets); CT indicated	R–R	All	173	173 (100%)	− 129 (− 75%)					
Malam 2022 [61] **UK** (2014–2020)	LN1–3; post-test decision based on RS	R–R	All	69	32 (46%)	− 19 (− 28%)					
Battisti 2019 (abst) [58] **UK** (2017–2018)	LN1–3	R–R	All	567	371 (65%)	− 209 (− 37%)					
		R–D	All	567	371 (65%)	− 231 (− 41%)					
Eiermann 2013 [69] **Germany** (2010–2011)	LN1–3	R–R	All	122	92 (75%)	− 22 (− 18%)					
		R–D	All	122	92 (75%)	− 35 (− 29%)					
Cognetti 2021 [63] **Italy** (2016–2017)	LN1–3	R–R	All	414	258 (62%)	− 148 (− 55%)					
Dieci 2018 [64] **Italy** (2014–2016)	LN1–3; intermediate clinical risk	R–R	All	126	72 (57%)	− 15 (− 12%)					
		R–D	All	126	72 (57%)	− 18 (− 14%)					
Fernandez-Perez 2021 (abst) [66] **Spain** (2013–2018)	LN1–3 (incl. micromets)	R–R	All	229	159 (69%)	− 100 (− 44%)					

*Hyphen* not reported, *Abst* abstract, *CT* chemotherapy, *D* decision, *ER* oestrogen receptor, *HER2* human epidermal growth factor receptor 2, *HR* hormone receptor positive, *LN* lymph nodes (number positive), *meno* menopausal, *micromets* micrometastases, *NR* not reported, *R* recommendation, *R–D* change from pre-test recommendation to post-test decision for chemotherapy, *R–R* change from pre-test recommendation to post-test recommendation for chemotherapy, *RS* Recurrence Score (Oncotype DX)

#### Decision impact results across all test risk groups

Across all test risk groups in all 12 studies [58–70], the net change in the percentage of patients with a chemotherapy recommendation or decision (pre- to post-test) was a reduction of 12% to 75%. Four studies had characteristics potentially influencing results as follows: three [60, 62, 67] only included patients for whom chemotherapy was indicated pre-test, and in two [60, 61] the post-test decision was based almost entirely on the RS score. Excluding these four studies, the net change in chemotherapy recommendation or decision across the remaining eight studies [58, 59, 63–66, 68–70] was a reduction of 12% to 57%. One study reported a greater reduction in chemotherapy decisions in post-menopausal patients (reduction of 57%) than pre-menopausal patients (reduction of 34%) [70].

#### Decision impact results by test risk group

Four studies presented data by Oncotype DX risk group using RS 18 and 30 cut-offs [59, 60, 67, 68, 70], and two studies using the RS 25 cut-off [59, 65, 70]. Across the four studies using RS 18 and 30 cut-offs, the net change in chemotherapy recommendations or decisions was: a reduction of 20% to 93% in the RS 0–17 risk group; a reduction of 19% to 54% in the RS 18–30 risk group; and between a 17% reduction (*n* = 1 patient) and a 2% increase in the RS > 30 risk group [59, 60, 67, 68, 70]. Excluding two studies with limitations as described above [60, 67], the net change in the remaining two studies [59, 65, 70] was: a reduction of 20% to 68% in the RS 0–17 risk group; a reduction of 19% to 35% in the RS 18–30 risk group; and no change or a 2% increase in the RS > 30 risk group, respectively.

In two studies using a cut-off of RS ≤ 25 [59, 65, 70], the net change in chemotherapy recommendations or decisions was: a reduction of 29% to 61% in the RS 0–25 risk group; and no change or an increase of 5% in the RS > 25 risk group, respectively.

### HRQoL and anxiety

No studies reported HRQoL or anxiety associated with use of GEP tests in LN+ populations. A brief summary of such studies in LN0 or mixed populations is provided in the Discussion.

## Discussion

### Prognostic and predictive ability: summary of findings and limitations

This systematic review summarises evidence in LN+ populations for prognostic ability, prediction of chemotherapy benefit, and effect on adjuvant chemotherapy decisions for four GEP tests. All four tests have some evidence of prognostic ability in LN+ populations. Studies assessing prediction of chemotherapy benefit in LN+ populations were only identified for Oncotype DX and MammaPrint. Overall, more published evidence specific to LN+ populations was identified for Oncotype DX than for the other three tests.

The RxPONDER RCT [16, 18] of Oncotype DX evaluated whether adjuvant chemotherapy could be avoided in LN+ populations with a lower genomic risk (RS 0–25). RxPONDER demonstrated no chemotherapy benefit in LN+ post-menopausal patients with RS 0–25, potentially suggesting that chemotherapy maybe avoided in these women. However, LN+ pre-menopausal patients had statistically significant chemotherapy benefit despite low Oncotype DX scores (RS 0–25). The MINDACT RCT [10] of MammaPrint reported that LN+ patients with high clinical risk and low MammaPrint risk (across all age groups) had a non-statistically significant benefit from chemotherapy. Similarly to RxPONDER, chemotherapy benefit was smaller in LN+ patients aged > 50 years. No data were reported for LN+ patients ≤ 50 years; however, there was a significant chemotherapy benefit in the ≤ 50 years subgroup as a whole (combined LN0/LN+ group). This potentially indicates less utility of GEP tests in pre-menopausal LN+ patients, as reflected in the ASCO 2022 guidelines [8] which do not recommend any GEP test in LN+ pre-menopausal patients.

RxPONDER was not designed to assess the relationship between RS and magnitude of chemotherapy benefit for RS > 25, although within the range RS 0–25, an interaction test between RS and chemotherapy effect was not statistically significant. Conversely, a reanalysis of SWOG-8814 [17] suggested that post-menopausal LN+ patients with higher Oncotype RS may experience a greater relative benefit of chemotherapy than those with lower RS. MINDACT was also not designed to assess differential chemotherapy benefit per risk group.

The majority (65%) of patients in RxPONDER had only 1 positive node (whilst 25% had 2 positive nodes and 9% had 3 positive nodes), although post-menopausal patients showed no significant effect of chemotherapy either in subgroups with 1 positive node or with 2–3 positive nodes [16]. Furthermore, patients screened for RxPONDER received their RS result before agreeing to randomisation, which may have resulted in selection bias (of 9383 women screened, 4300 were excluded before randomisation, of which 1035 had RS > 25 but the remaining 3265 did not participate for other reasons). In general, some selection bias may be present in any prospective study of GEP tests, since patients with fewer clinical risk factors may be more likely to participate. In addition, it has been suggested that the benefits of adjuvant chemotherapy in pre-menopausal women may be related to chemotherapy-induced ovarian function suppression. Benefits of chemotherapy in pre-menopausal women (or those aged < 50 years) have been reported in RxPONDER (Oncotype DX, LN+ patients) [16], MINDACT (MammaPrint, combined LN0/LN+ group) [10] and TAILORx (Oncotype DX, LN0 patients), with some analyses showing a late benefit of chemotherapy in women close to menopause. If this is the case, it may be possible to spare chemotherapy in some pre-menopausal women in favour of optimising endocrine therapy and ovarian function suppression.

There are major challenges when designing studies to assess prediction of chemotherapy benefit, due to ethical issues with randomising genomic high-risk patients to receive chemotherapy or no chemotherapy. This is because, based on prognostic ability, the absolute risk of recurrence (and absolute chemotherapy benefit) is greater for high-risk patients, irrespective of relative chemotherapy benefit. Overall, the ability of GEP tests to predict chemotherapy benefit in LN+ patients remains uncertain.

### Decision impact: summary of findings and limitations

Impact on chemotherapy decisions for LN+ populations in the UK and Europe was only reported for Oncotype DX. Recommendations or decisions to undergo chemotherapy following Oncotype DX testing reduced by 12–75% across all 12 studies [58–70], or 12–57% across 8 studies more representative of clinical practice [58, 59, 63–66, 68–70]. There were greater reductions in groups with lower RS [59, 60, 65, 67, 68, 70]. The absence of decision impact studies for EndoPredict, Prosigna and MammaPrint represents a gap in the current evidence base.

### Anxiety and HRQoL: summary of findings and limitations

Our previous review [6] identified studies assessing anxiety or HRQoL for all four tests in LN0 or mixed nodal status populations (an update search identified no additional studies). Some studies reported significant improvements in anxiety after testing, whilst others reported no significant change, and some reported a decrease in anxiety after a low-risk result or when treatment was downgraded to no chemotherapy, but an increase in anxiety after a high-risk result or when treatment was upgraded to chemotherapy [6]. No studies of anxiety or HRQoL impact of testing in LN+ populations were identified. Nonetheless, these are key considerations for clinicians and patients during shared decision-making regarding chemotherapy, therefore more research is warranted.

### Clinical implications

Use of testing in LN+ populations would likely lead to more patients in low-risk groups avoiding chemotherapy and associated adverse effects. However, unless chemotherapy offers zero benefit to low-risk patients, some people who avoid chemotherapy following testing may subsequently develop cancer recurrence. The four tests allocate differing numbers of people to risk groups, based on reanalyses of trials and cohorts. Prosigna and EndoPredict allocate fewer LN+ patients to low-risk groups, which have quite favourable 10-year DRFIs (Table 1). Conversely, Oncotype DX and MammaPrint allocate more LN+ patients to low-risk groups (with registry data suggesting an Oncotype DX cut-off of RS ≤ 25 would allocate 80–90% to the low-risk group), and 10-year distant recurrence for low-risk patients is less favourable for Oncotype DX and MammaPrint, based on prognostic studies. If a test was predictive of chemotherapy benefit, it might be assumed that low-risk patients would not benefit from chemotherapy, irrespective of their absolute recurrence risk. There is some evidence for Oncotype DX being predictive of chemotherapy benefit in LN+ post-menopausal patients, but this remains uncertain. It was not possible to determine whether MammaPrint was predictive for chemotherapy benefit.

### Suggested research priorities

Further studies evaluating statistical interactions between test group and effect of chemotherapy may help address uncertainty around predictive benefit. However, prospective studies may be difficult to ethically design, whilst observational and registry studies may provide these data but with risks of confounding. Studies assessing decision impact for Prosigna, EPclin and MammaPrint in LN+ patients would be valuable. Since decision tools based on clinico-pathological features also have prognostic ability, the integration of decision aid tools with GEP tests to support shared decision-making may constitute a useful research direction [71]. The role of gene testing in older adults, who may be more prone to chemotherapy complications in the context of limited life expectancy and reduced treatment benefits, is also a research priority [72].

## Conclusions

Oncotype DX, Prosigna, EndoPredict and MammaPrint have prognostic ability in LN+ patients. Evidence on predictive benefit is weaker, though post-menopausal LN+ patients with low Oncotype DX scores may have reduced benefit from chemotherapy. Use of Oncotype DX in practice leads to fewer patients being recommended chemotherapy.

## Supplementary Information

Below is the link to the electronic supplementary material.

Supplementary file1 (DOCX 537 KB)

## Data Availability

No datasets were generated or analysed during the current study.
