# Supplementary material for: Gene expression profiling tests to guide adjuvant chemotherapy decisions in lymph node-positive early breast cancer: a systematic review
Source: Breast Cancer Res Treat. 2025 Feb 3;210(2):229–47. doi: 10.1007/s10549-024-07596-0 (PMC11930876; doi:10.1007/s10549-024-07596-0)
Supplement: Supplementary file 1 — Supplementary file1 (DOCX 537 KB) [file 10549_2024_7596_MOESM1_ESM.docx]

**Gene expression profiling tests to guide adjuvant chemotherapy decisions in lymph node-positive early breast cancer: A systematic review**

**Authors:** Katy Cooper^a^, Gamze Nalbant^a^, Munira Essat^a^, Sue Harnan^a^, Ruth Wong^a^, Jean Hamilton^a^, Uzma S. Asghar^b^, Nicolò M. L. Battisti^c^, Lynda Wyld^a^, Paul Tappenden^a^.

# **APPENDICES**

**Appendix A: Literature search strategy**

**Sources searched**

The databases, trial registers and websites searched included the following:

- MEDLINE and MEDLINE in Process (via Ovid)
- EMBASE (via Ovid)
- Cochrane Database of Systematic Reviews (via Wiley)
- Cochrane Central Register of Controlled Trials (via Wiley)
- HTA Database of the International Network of Agencies for Health Technology Assessment (INAHTA)
- Web of Science Citation Index Expanded (via Clarivate)
- Web of Science Conference Proceedings Citation Index (via Clarivate)
- World Health Organization International Clinical Trials Registry Platform (WHO ICTRP)
- Clinicaltrials.gov (National Library of Medicine)
- American Society of Clinical Oncology (ASCO)
- European Society for Medical Oncology (ESMO)
- American Association for Cancer Research (AACR)
- European Cancer Organization (ECO).

**Search strategy**

**Ovid MEDLINE(R) and Epub Ahead of Print, In-Process, In-Data-Review & Other Non-Indexed Citations and Daily 1946 to April 25, 2023**

| **#** | **Searches** |
| --- | --- |
| 1 | exp Breast Neoplasms/ |
| 2 | exp mammary neoplasms/ |
| 3 | exp breast/ |
| 4 | exp neoplasms/ |
| 5 | 3 and 4 |
| 6 | (breast* adj5 (neoplasm* or cancer* or tumo?r* or carcinoma* or adenocarcinoma* or sarcoma* or dcis or ductal or infiltrat* or intraductal* or lobular or medullary)).mp. |
| 7 | (mammar* adj5 (neoplasm* or cancer* or tumo?r* or carcinoma* or adenocarcinoma* or sarcoma* or dcis or ductal or infiltrat* or intraductal* or lobular or medullar)).mp. |
| 8 | 1 or 2 or 5 or 6 or 7 |
| 9 | (endopredict or epclin or "ep score").mp. |
| 10 | (mammaprint or 70-gene or "70 gene").mp. |
| 11 | (oncotype or "recurrence score" or 21-gene or "21 gene").mp. |
| 12 | (prosigna or pam50 or 50-gene or "50 gene").mp. |
| 13 | or/9-12 |
| 14 | 8 and 13 |
| 15 | limit 14 to yr="2017 -Current" |

Search strategy adapted from Harnan et al., (2019) © Queen’s Printer and Controller of HMSO 2019.

**Appendix B: PRISMA flow diagram**

**Identification of studies via databases and registers**

Records identified from: (n=7,039)

MEDLINE and Medline in Process (n = 1,192)

Embase (n = 3,184)

Web of Science (n = 1,846)

Cochrane CENTRAL (n = 507)

Cochrane CDSR (n = 132)

Trial registries (n = 101)

INAHTA (n = 77)

Studies identified from Harnan *et al.*

(n=13)

Records removed *before screening*:

Duplicate records removed (n = 2,981)

**Identification**

Records excluded at title/abstract sift (n = 3,556)

Records screened

(n = 4,058)

Reports not retrieved

(n = 0)

Reports sought for retrieval

(n = 502)

**Screening**

Reports excluded (n = 460):

Conference abstract pre-2021 (n = 162)

Non-relevant outcomes (n = 112)

Non-relevant population: not LN+ (n = 63)

Non-relevant study design (n = 35)

Non-relevant test (n = 19)

Non-relevant population: other (n = 18)

Decision impact non-Europe (n = 15)

Superceded by other reference (n = 13)

No results reported (n = 12)

Not correct version of test (n = 6)

Not English language (n = 1)

Already included from DG34 (n = 4)

Reports assessed for eligibility

(n = 502)

**Included**

Reports included from search

(n = 42)

Reports included from previous review (n = 13)

Total reports included (n = 55)

*From:*  Page MJ, McKenzie JE, Bossuyt PM, Boutron I, Hoffmann TC, Mulrow CD, et al. The PRISMA 2020 statement: an updated guideline for reporting systematic reviews. BMJ 2021;372:n71. doi: 10.1136/bmj.n71

For more information, visit: <http://www.prisma-statement.org/>

**Appendix C: Risk of bias assessment**

*Risk of bias assessment strategy*

Studies were assessed using risk of bias assessment tools relevant to the study design. Prospective RCTs were assessed using the Cochrane Risk of Bias tool (RoB2) [14]. Prognostic and prediction studies were assessed using the Prediction model study Risk Of Bias Assessment Tool (PROBAST) [15]; items from each domain were selected based on their relevance to this review, and definitions of high or low risk for each item specific to this review were defined a priori. Each study, cohort or registry was assessed once, rather than assessing each publication separately. Decision impact studies did not undergo formal quality assessment, but design and relevance were considered narratively. The impact of the quality of studies on the evidence base was considered within the narrative synthesis.

*Definition of items in PROBAST for this review*

For assessment of prognostic and prediction studies, items from each domain of PROBAST were selected based on their relevance to this review, and definitions of high or low risk for each item specific to this review were defined a priori, as shown in Table S1.

**Table C.1: Risk of bias and applicability (adapted from PROBAST)**

| **Risk of bias** |  |  |
| --- | --- | --- |
| **Domain** | **Criterion** | **Scoring for this review** |
| Domain 1 Participants | Were appropriate data sources used? | - Yes (prognosis): reanalysis of RCT or cohort or nested case control AND patients did not receive chemotherapy - Yes (predicting chemotherapy benefit): RCT or reanalysis of RCT - No (prognostic): non-nested case control or case series AND/OR some/all patients had chemotherapy - No (predicting chemotherapy benefit): patients not randomised to chemotherapy vs. no chemotherapy |
| Domain 1 Participants | Were all inclusions and exclusions of participants appropriate? | - Yes: all eligible patients from trial or consecutive eligible patients from prospective registry - No: some eligible patients excluded (e.g. not sent for testing, insufficient tissue, test failures, missing data, AND/OR non-prospective registry) - Unclear: if unclear |
| Domain 2 Predictors [tests] | Were the tests [predictors] defined and assessed in a similar way for all participants? | - Yes: If test assessed in similar way for all participants [most/all studies in this review likely to score Yes as uses standardised test] - No: Test not assessed in similar way for all participants |
| Domain 2 Predictors [tests] | Were the tests [predictor assessments] made without knowledge of outcome data? | - Yes: If test assessors blinded to clinical outcomes - No: If not blinded - Unclear: if unclear |
| Domain 3 Outcomes | Were the outcome definitions standardised or defined a priori? | - Yes: At least one outcome was standardised (e.g. DRFS, OS) or defined a priori - No: All outcomes non-standardised and not defined a priori - Unclear: if unclear |
| Domain 3 Outcomes | Were the outcomes determined without knowledge of test [predictor] information? | - Yes: If outcome assessors blinded to test results - No: If not blinded - Unclear: if unclear |
| Domain 3 Outcomes | Was chemotherapy decision made before test result known? | - Yes: Test did not influence use of chemotherapy [Yes if retrospective use of test on stored tumour samples, i.e. reanalyses of RCTs or cohorts] - No: Test result may have influenced use of chemotherapy [No for observational studies of prospective use of test] - [This item is not in PROBAST but is important for this review] |
| Domain 4 Analysis | Were there a reasonable number of participants with outcome data? | - Yes: At least 100 patients with outcome data - No: Less than 100 patients with outcome data |
| Domain 4 Analysis | Were all enrolled participants included in the analysis? | - Yes: If all enrolled participants included in the analysis - No: If some enrolled patients not analysed |
| **Applicability** |  |  |
| **Number** | **Criterion** | **Scoring for this review** |
| Domain 1 Participants | Did the included participants match the review question? | - Yes: all patients in scope (HR+, HER2–, LN1–3) - Mostly: < 20% out of scope - No: > 20% out of scope - Unclear: if unclear |
| Domain 2 Predictors [tests] | Did the definition and assessment of tests [predictors] match the review question? | - Yes: same as commercially available tests - No: different from commercially available tests (e.g. FFPE vs. fresh samples, test methods) |
| Domain 3 Outcomes | Did the outcomes match the review question? | - Yes: At least one outcome matched the review question - No: No outcomes matched the review question |

*Results: risk of bias in prospective RCTs*

The risk of bias in the two prospective RCTs, assessed using the Cochrane RoB2 tool [14], is shown in Table S2. The two RCTs scored low risk of bias on all domains, and low risk of bias overall.

**Table C.2: Risk of bias in prospective RCTs (using Cochrane RoB2)**

| **RCT** | **Risk of bias due to …** | | | | | |
| --- | --- | --- | --- | --- | --- | --- |
|  | **Randomisation process** | **Deviations from intended interventions** | **Missing outcome data** | **Measurement of the outcome** | **Selection of the reported result** | **Overall risk of bias** |
| RxPONDER  Kalinsky 2021 [16] | Low | Low | Low | Low | Low | **Low** |
| MINDACT  Piccart 2021 [10] | Low | Low | Low | Low | Low | **Low** |

*Results: risk of bias in prognostic studies*

The risk of bias in prognostic studies, assessed using the PROBAST tool [15], is presented in Table S3 for RCT reanalyses and cohort reanalyses (within which the test was used retrospectively), and in Table S4 for observational studies (within which the test was used prospectively).

The following factors may have affected results to some extent. For Domain 1 (participants), studies varied in terms of whether participants received chemotherapy or not; studies are therefore reported separately according to chemotherapy use in the section on prognostic ability (Section 3.4). In some studies, some participants did not match the review question (either not ER+, not HER2- or not LN1-3); these factors were taken into account when selecting studies for use in the economic model. Most studies excluded a proportion of patients for various reasons including insufficient tissue, missing data, failed tests and others, which may have influenced results to some extent, though the impact is difficult to assess. For Domain 3 (outcomes), chemotherapy decisions were not influenced by the test result in studies of retrospective use of the test (i.e., reanalyses of RCTs and cohorts), whereas in observational studies in which the test was used prospectively, chemotherapy decisions may have been influenced by the test result; therefore, observational studies are reported separately in the section on prognostic ability (Section 3.4).

The following factors were either judged low risk or were unlikely to have affected results. For Domain 2 (predictors, i.e., the tests themselves), all studies used the same version of the test for all participants (as the tests are standardised). Some studies blinded test assessors to patient outcomes while for other studies this was unclear; however, since the tests are based on objective measures of gene expression, this is unlikely to have affected interpretation of test results. For Domain 3 (outcomes), all studies used standardised outcomes relating to recurrence or survival. It was assumed that blinding of outcome assessors to test results applied within studies of retrospective use of the test, while in studies of prospective use, blinding to test results was generally unclear; however, as most outcomes were standardised cancer outcomes, this is unlikely to have affected outcome reporting. For Domain 4 (analysis), most studies included a reasonable number of participants (over 100). In terms of applicability to the review question, the test and outcomes matched the review question in all studies.

**Table C.3: Risk of bias in prognostic studies (retrospective reanalyses of RCTs and cohorts)**

| **Reference** | **Cohort** | **Design**  **Derivation or validation?** | **Risk of Bias** | | | | | | | | | **Applicability** | | |
| --- | --- | --- | --- | --- | --- | --- | --- | --- | --- | --- | --- | --- | --- | --- |
|  |  |  | **Domain 1 Participants** | | **Domain 2 Predictors** | | **Domain 3 Outcomes** | | | **Domain 4 Analysis** | | **Participants** | **Predictors** | **Outcomes** |
|  |  |  | **Appropriate data sources?** | **Appropriate exclusions?** | **Tests same for all participants?** | **Blinded test assessors to outcomes?** | **Outcomes**  **standardised**  **or a priori?** | **Blinded outcome assessors to test?** | **CT decision made before test result known?** | **Participants N>100?** | **All analysed?** | **Participants match review question?** | **Tests match review question?** | **Outcomes match review question?** |
| Albain 2010 [17] | SWOG-8814 | RCT-R  V | Y (RCT-R, ET only) | N (InT, TF) | Y | Y | Y | Y | Y | Y | N | N (>20%  LN4+) | Y | Y |
| Constantinidou 2022 [30] | Cyprus + Notts | Cohort-R  V | Y (cohort-R, ET only) | N (InT, MD) | Y | Y | Y | Y | Y | N | N | Y | Y | Y |
| Drukker 2014 [22] | VdV cohort , Netherlands | Cohort-R  V (21% also  in derivation  set) | N (cohort-R,  some CT) | Y | Y | UC | Y | Y | Y | Y | Y | N (>20% ER- and >20% LN4+) | Y | Y |
| Filipits 2019 [29] | ABCSG-6/8 | RCT-R  V | Y (RCT-R, ET only) | UC | Y | UC | Y | Y | Y | Y | Y | Y | Y | Y |
| Gnant 2014 [24] Filipits 2014[25] | ABCSG-8, Austria | RCT-R  V | Y (RCT-R, ET only) | N (InT, MS, TF, no consent) | Y | Y | Y | Y | Y | Y | N | Mostly (11% LN4+) | Y | Y |
| Jackisch 2022 (abst) [33] | Germany, PATH | Cohort-R  V | N (cohort-R, some CT) | N (reason NR) | Y | UC | Y | Y | Y | N | N | UC | Y | Y |
| Laenkholm 2018 [26] | DBCG, Denmark | Cohort-R  V | Y (cohort-R, ET only) | N (FT, MD) | Y | Y | Y | Y | Y | Y | N | Y | Y | Y |
| Lundgren 2022 [35] | SBII:pre trial | RCT-R  V | Y (RCT-R, ET only) | N (InT, FT, MD) | Y | N | Y | Y | Y | Y | Y | Y | Y | Y |
| Mamounas 2018 [21] | NSABP-28 | RCT-R  V | N (RCT-R, all CT) | N (InT, MS) | Y | UC | Y | Y | Y | Y | Y | N (HER2 NR) | Y | Y |
| Martin 2016 [27], Martin 2014 [28] | GEICAM 9906, Spain | RCT-R  V | N (RCT-R, adjuvant CT) | N (MD) | Y | Y | Y | Y | Y | Y | N | N (>20% LN4+) | Y | Y |
| Mook 2009 [19] | NKI and Italy | Cohort-R  V | N (cohort-R,  some CT) | N (InT, RNA quality) | Y | Y | Y | Y | Y | Y | Y | N (>20% ER-,  16% HER2+) | Y | Y |
| Penault-Llorca 2018 [32] | PACS01 | RCT-R  V | N (RCT-R, some CT) | N (FT, InT, MS) | Y | Y | Y | Y | Y | Y | Y | N (>20%  LN4+) | Y | Y |
| Pu 2020 [34] | WHEL Study | RCT-R | N (RCT-R, some CT) | N (InT, MS, TF) | Y | UC | Y | Y | Y | Y | N | UC (NR N nodes) | Y | Y |
| Sestak 2018 [20], 2017 [37] | TransATAC | RCT-R  V | Y (RCT-R, ET  only) | N (InT; FT ) | Y | Y | Y | Y | Y | Y | Y | Y | Y | Y |
| Sestak 2020 [36] | Lobular subgroup (TransATAC + ABCSG-6/8) | RCT-R  V | Y (RCT-R, ET only) | UC | Y | Y | Y | Y | Y | Y | N | Mostly (20% LN4+) | Y | Y |
| Vliek 2017 [23] | RASTER | Cohort-R  V | N (cohort-R, some CT) | N (InT, MS, no consent) | Y | UC | Y | Y | Y | Y | Y | Mostly (17% ER-, 15% HER2+) | Y | Y |

*Cohort-R - reanalysis of cohort study; CT - chemotherapy; D - development study; ET - endocrine therapy; FT - failed test; InT - insufficient tissue; MD - missing data; MS - missing samples; N - no; NR - not reported; LN - number of positive lymph nodes; RCT-R - reanalysis of RCT; TF - test failure; UC - unclear; V - validation study; Y - yes*

**Table C.4: Risk of bias in prognostic studies (observational studies of prospective use of test)**

| **Reference** | **Cohort** | **Design**  **Derivation or validation?** | **Risk of Bias** | | | | | | | | | **Applicability** | | |
| --- | --- | --- | --- | --- | --- | --- | --- | --- | --- | --- | --- | --- | --- | --- |
|  |  |  | **Domain 1 Participants** | | **Domain 2 Predictors** | | **Domain 3 Outcomes** | | | **Domain 4 Analysis** | | **Participants** | **Predictors** | **Outcomes** |
|  |  |  | **Appropriate data sources?** | **Appropriate exclusions?** | **Tests same for all participants?** | **Blinded test assessors to outcomes?** | **Outcomes**  **standardised**  **or a priori?** | **Blinded outcome assessors to test?** | **CT decision made before test result known?** | **Participants N>100?** | **All analysed?** | **Participants match review question?** | **Tests match review question?** | **Outcomes match review question?** |
| Braun 2022 [51] | Red Cross Hospital, Munich, Germany | Observational  V | N (prospective use of test, some CT) | Y | Y | Y | Y | UC | N | Y | N | Mostly (20% LNmic) | Y | Y |
| Ibraheem 2020 [40] | NCDB | Observational  V | N (prospective use of test, CT) | N (MD, SFT) | Y | Y | Y | UC | N | Y | Y | Y | Y | Y |
| Massarweh 2018 [47]  Petkov 2016 [45]  Roberts 2017 [46] | SEER | Observational  V | N (prospective use of test, some CT) | N (InT, MS, SFT, no consent) | Y | Y | Y | Y | N | Y | Y | UC (% LNmic NR) | Y | Y |
| Nitz 2017 [50] | WSG PlanB | Observational  V | N (prospective use of test, some CT) | N (dropout, screening failure) | Y | Y | Y | UC | N | Y | Y | Y | Y | Y |
| Poorvu 2020 [49] | Young Women's Breast Cancer Study | Observational  V | N (part prospective use of test, part stored samples, some CT) | N (InT, MS, no consent) | Y | Y | Y | UC | N | Y | N | UC (% LNmic NR) | Y | Y |

*CT - chemotherapy; D - development study; InT - insufficient tissue; MD - missing data; MS - missing samples; N - no; NCDB - National Cancer Database; NR - not reported; LN - number of positive lymph nodes; SFT - only those sent for test included; UC - unclear; V - validation study; WSG - West German Study Group; Y - yes*

*Results: risk of bias in prediction studies*

The risk of bias in prediction studies, assessed using the PROBAST tool [15], is presented in Table S5.

The following factors may have affected results to some extent. For Domain 1 (participants), only the SWOG-8814 study [17] was a reanalysis of an RCT in which chemotherapy use was randomised; in the remaining studies, chemotherapy use was not randomised. This limitation is reflected in the section on prediction of chemotherapy benefit (Section 3.5). In some studies, some participants did not match the review question (either not ER+, not HER2- or not LN1-3). Most studies excluded a proportion of patients for various reasons including insufficient tissue, missing data, failed tests and others, which may have influenced results to some extent, though the impact is difficult to assess. For Domain 3 (outcomes), chemotherapy decisions were not influenced by the test result in the two studies of retrospective use of the test, whereas in the three observational registries in which the test was used prospectively, chemotherapy decisions may have been influenced by the test result; therefore, observational studies are reported separately in the section on prediction of chemotherapy benefit (Section 3.5).

The following factors were either judged low risk or were unlikely to have affected results. For Domain 2 (predictors, i.e., the tests themselves), all studies used the same version of the test for all participants (as the tests are standardised), and all studies blinded test assessors to patient outcomes. For Domain 3 (outcomes), all studies used standardised outcomes relating to recurrence or survival, and in all studies outcome assessors were blinded to test results. For Domain 4 (analysis), all studies included a reasonable number of participants (over 100). In terms of applicability to the review question, the test and outcomes matched the review question in all studies.

**Table C.5: Risk of bias in prediction studies**

| **Reference** | **Cohort** | **Derivation or validation?** | **Risk of Bias** | | | | | | | | | **Applicability** | | |
| --- | --- | --- | --- | --- | --- | --- | --- | --- | --- | --- | --- | --- | --- | --- |
|  |  |  | **Domain 1 Participants** | | **Domain 2 Predictors** | | **Domain 3 Outcomes** | | | **Domain 4 Analysis** | | **Participants** | **Predictors** | **Outcomes** |
|  |  |  | **Appropriate data sources?** | **Appropriate exclusions?** | **Tests same for all participants?** | **Blinded test assessors to outcomes?** | **Outcomes**  **standardised**  **or a priori?** | **Blinded outcome assessors to test?** | **CT decision made before test result known?** | **Participants N>100?** | **All analysed?** | **Participants match protocol?** | **Tests match review question?** | **Outcomes match review question?** |
| Albain 2010 [17] | SWOG-8814 | RCT-R  V | Y (RCT-R) | N (InT, TF) | Y | Y | Y | Y | Y | Y | N | N (>20%  LN4+) | Y | Y |
| Mook 2009 [19] | NKI and Italy | Cohort-R  V | N (not RCT) | N (InT, RNA qual) | Y | Y | Y | Y | Y | Y | Y | N (>20% ER-, 16% HER2+) | Y | Y |
| Abel 2022 [54]  Cao 2022 (abst) [55]  Ibraheem 2019 [41]  Iorgulescu 2019 [56]  Kumar 2023 (abst) [57]  Nash 2023 [42]  Weiser 2021 [44]  Weiser 2022 [43] | NCDB | Observational  V | N (not RCT) | N (MD, SFT) | Y | Y | Y | Y | N | Y | N | Y | Y | Y |
| Petkov 2020 (abst) [53] | SEER | Observational  V | N (not RCT) | N (InT, MS, SFT, no consent) | Y | Y | Y | Y | N | Y | Y | UC (% LNmic NR) | Y | Y |
| Rotem 2022 (abst) [52]  Stemmer 2017 [48] | Clalit, Israel | Observational  V | N (not RCT) | N (SFT) | Y | Y | Y | Y | N | Y | N | N (>20% LNmic) | Y | Y |

*Cohort-R - reanalysis of cohort study; D - development study; InT - insufficient tissue; MD - missing data; MS - missing samples; NCDB - National Cancer Database; N - no; NR - not reported; LN - number of positive lymph nodes; LNmic - lymph node micrometastases; RCT-R - reanalysis of RCT; SFT - only those sent for test included; TF - test failure; UC - unclear; V - validation study; Y - yes*

**Appendix D: Additional tables for prognostic ability**

**Table D.1: Prognostic data (Oncotype DX)**

| **Reference**  **Study/cohort** | **Outcome** | **N, ET/CT**  **Design** | **Nodal status**  **HR, HER2** | **Meno**  **status** | **Test cut-offs** | **Distribution %** | | | **Risk 0-5yr %** | | | **Risk 0-10yr/other %** | | | **HR between test groups (95% CI)** | **^a^Sig?**  ***Adj** |  |
| --- | --- | --- | --- | --- | --- | --- | --- | --- | --- | --- | --- | --- | --- | --- | --- | --- | --- |
|  |  |  |  |  |  | **Low** | **Int** | **High** | **Low** | **Int** | **High** | **Low** | **Int** | **High** |  |  |  |
| **Oncotype DX: Distant recurrence, ET monotherapy** | | | | | | | | | | | | | | | | | |
| Sestak 2018 [20] 2017 [37]  TransATAC | DRFI | n=183  ET mono  RCT-R | LN1-3  100% HR+ 100% HER2- | Post-meno | 18, 30 | 57 | 32 | 11 | 95.9 | 84.8 | 83.6 | 0-10y 80.6  5-10y 82.1 | 0-10y 70.9  5-10y 80.5 | 0-10y 62.0  5-10y 72.5 | 0-5yr: Int vs. low: HR 3.84 (1.31 to 11.23) 0-5yr: High vs. low: HR 4.45 (1.19 to 16.58)  0-10yr: Int vs. low: HR 1.66 (0.86 to 3.23)  0-10yr: High vs. low: HR 2.35 (0.99 to 5.60) 0-10yr: Per 1SD change: 1.39 (1.05-1.85)  *Adj: LR vs. CTS (*p*=0.06) and NPI (*p* = 0.1) | Y  Y  N  N  Y  N* |  |
| **Oncotype DX: Distant recurrence, variable ET/CT** | | | | | | | | | | | | | | | | | |
| Mamounas 2018 [21]  NSABP-28 | DRFI | n=722  All CT+ET RCT-R | LN1-3  100% ER+ NR HER2 | All meno | 18, 30 | 37 | 34 | 28 | - | - | - | 84.7 | 71.5 | 63.1 | 0-10yr: *p*<0.001  *0-10yr: Adj HR per 50-RS: 2.42 (NR); *p*<0.001 | Y  Y* |  |
| Penault-Llorca 2018 [32]  PACS01 | DRFI | n=530  All CT  74% ET  RCT-R | LN1-3: 60% LN4+: 40%  100% HR+ 90% HER2- | All meno (39% post) | 18, 30 | 39 | 30 | 31 | 93.7 | 87.3 | 69.3 | - | - | - | 0-5yr: HR per 50-RS: 4.14 (2.67 to 6.43); *p*<0.001  *0-5yr: Adj HR 3.36 (1.88 to 6.00), *p*<0.001 | Y  Y* |  |
| **Oncotype DX: DFS** | | | | | | | | | | | | | | | | | |
| Albain 2010 [17]  SWOG-8814 | DFS | n=148  ET mono  RCT-R | LN+ 100% LN4+: 37%  100% HR+, 91% HER2– | Post-meno | 18, 30 | 37 | 31 | 32 | - | - | - | 60 | 49 | 43 | 0-5yr: HR 5.55 (2.32 to 3.28); *p*=0.0002  0-10yr: Between risk groups: *p*=0.017  0-10yr: HR per 50-RS: 2.64 (1.33 to 5.27); *p*=0.006  5-10yr: HR 0.86 (0.27 to 2.74); *p*=0.80 | Y  Y  Y  N |  |
| Mamounas 2018 [21]  NSABP-28 | DFS | n=722  All CT+ET RCT-R | LN1-3  100% ER+ NR HER2 | All meno | 18, 30 | 37 | 34 | 28 | - | - | - | 79.8 | 64.8 | 57 | 0-10yr: *p*<0.001  *0-5yr: Adj HR per 50-RS 3.81 (2.67 to 5.43); *p*<0.001 *0-10yr: Adj HR per 50-RS 2.53 (1.90 to 3.38); *p*<0.001 *5-10yr: Adj HR per 50-RS 1.39 (0.88 to 2.19); *p*=0.16 | Y  Y*  Y*  N* |  |
| Penault-Llorca 2018 [32]  PACS01 | DFS | n=530  All CT  74% ET  RCT-R | LN1-3: 60% LN4+: 40%  100% HR+ 90% HER2- | All meno (39% post) | 18, 30 | 39 | 30 | 31 | 90.8 | 84.9 | 64.6 | - | - | - | 0-5yr: HR per 50-RS: 3.28 (2.18 to 4.94); *p*<0.001  *0-5yr: Adj HR 2.66 (1.62 to 4.37), *p*<0.001 | Y  Y* |  |
| Kalinsky 2021 [16]  RxPONDER | IDFS | n=5,018  CT+ET vs. ET  Prosp RCT | LN1-3  100% HR+  100% HER2- | All meno (67% post) | All ≤25 | - | - | - | See prediction tables for outcomes per risk group | | | - | - | - | *0-5yr: HR per unit-RS (within RS 0-25): 1.05 (1.04 to 1.07), *p*<0.001 (adj meno and CT) | Y* |  |
|  |  |  |  | Post-meno | All ≤25 | - | - | - | - | - | - | - | - | - | *0-5yr: HR per unit-RS (within RS 0-25): 1.05 (1.03 to 1.07), *p*<0.001 (adj CT, nodes, grade, tumour size, age) | Y* |  |
|  |  |  |  | Pre-meno | All ≤25 | - | - | - | - | - | - | - | - | - | *0-5yr: HR per unit-RS (within RS 0-25): 1.06 (1.02 to 1.09), *p*=0.001 (adj CT, nodes, grade, tumour size, age) | Y* |  |
| Abdou 2023 [38]  RxPONDER | IDFS | n=4,015  CT+ET vs. ET  Prosp RCT | LN1-3  100% HR+  100% HER2- | White  n=2,833 | All ≤25 | - | - | - | 91.5 | |  | - | - | - | - | - |  |
|  |  |  |  | Black  n=248 | All ≤25 | - | - | - | 87.0 | |  | - | - | - | - | - |  |
|  |  |  |  | Asian  n=324 | All ≤25 | - | - | - | 93.9 | |  | - | - | - | - | - |  |
|  |  |  |  | Hispanic  n=610 | All ≤25 | - | - | - | 91.4 | |  | - | - | - | - | - |  |
| **Oncotype DX: OS and BCSS** | | | | | | | | | | | | | | | | | |
| Albain 2010 [17]  SWOG-8814 | OS | n=148  ET mono  RCT-R | LN+ 100% LN4+: 37%  100% HR+, 91% HER2– | Post-meno | 18, 30 | 37 | 31 | 32 | - | - | - | 77 | 68 | 51 | 0-10yr: Between risk groups: p=0.003  0-10yr: HR per RS-50: 4.42 (1.96 to 9.97), *p*=0.0006 | Y  Y |  |
| Penault-Llorca 2018 [32]  PACS01 | OS | n=530  All CT  74% ET  RCT-R | LN1-3: 60% LN4+: 40%  100% HR+ 90% HER2- | All meno (39% post) | 18, 30 | 39 | 30 | 31 | 99 | 95.6 | 85.6 | - | - | - | 0-5yr: HR per 50-RS: 5.0 (3.01 to 8.28); *p*<0.001 | Y |  |
| Mamounas 2018 [21]  NSABP-28 | OS | n=722  All CT+ET RCT-R | LN1-3  100% ER+ NR HER2 | All meno | 18, 30 | 37 | 34 | 28 | - | - | - | 93.3 | 79.2 | 70.7 | 0-10yr: p<0.001  *0-10yr: Adj HR per 50-RS: 3.09 (CI NR); *p*<0.001 | Y  Y* |  |
| Mamounas 2018 [21]  NSABP-28 | BCSS | n=722  All CT+ET RCT-R | LN1-3  100% ER+ NR HER2 | All meno | 18, 30 | 37 | 34 | 28 | - | - | - | 98 | 82.9 | 75.6 | 0-10yr: p<0.001  *0-10yr: Adj HR per 50-RS: 3.38 (CI NR); *p*<0.001 | Y  Y* |  |

*^a^The last column indicates whether each hazard ratio between test risk groups is statistically significant at the 5% level. Asterisk (*) denotes analyses adjusted for clinical factors.*

*Adj - adjusted; BCSS - breast cancer-specific survival; CI - confidence interval; CT - chemotherapy; CTS -* *Clinical Treatment Score (set of clinical factors); DFS - disease-free survival; DRFI - distant recurrence-free interval; ER - oestrogen receptor; ET - endocrine therapy; HER2 - human epidermal growth factor receptor 2; HR - hazard ratio; HR - hormone receptor; IDFS - invasive disease-free survival; int - intermediate; LN - lymph nodes (number positive); LR - likelihood ratio; meno - menopausal; NPI - Nottingham Prognostic Index; NR - not reported; OS - overall survival; prosp - prospective; RCT - randomised controlled trial; RCT-R - RCT reanalysis; RS - Recurrence Score (Oncotype DX); SD - standard deviation; sig - significant; y/yr - year*

**Table D.2: Prognostic data (MammaPrint)**

| **Reference**  **Study/cohort** | **Outcome** | **N, ET/CT**  **Design** | **Nodal status**  **HR, HER2** | **Meno**  **status** | **Test cut-offs** | **Distribution %** | | | **Risk 0-5yr %** | | | **Risk 0-10yr/other %** | | | **HR between test groups (95% CI)** | **^a^Sig?**  ***Adj** |  |
| --- | --- | --- | --- | --- | --- | --- | --- | --- | --- | --- | --- | --- | --- | --- | --- | --- | --- |
|  |  |  |  |  |  | **Low** | **Int** | **High** | **Low** | **Int** | **High** | **Low** | **Int** | **High** |  |  |  |
| **MammaPrint: Distant recurrence, ET monotherapy** | | | | | | | | | | | | | | | | | |
| No studies |  |  |  |  |  |  |  |  |  |  |  |  |  |  |  |  |  |
| **MammaPrint: Distant recurrence, variable ET/CT** | | | | | | | | | | | | | | | | | |
| Piccart 2021 [10]  MINDACT  (Not on prognostics summary table since CT use per risk group was influenced by test result) | DMFS | n=1,176  CT+ET vs. ET  Prosp-RCT | LN1-3  100% HR+  100% HER2- | High mAOL (n=989) | >0 low, ≤0 high | 69 | - | 31 | 95.7  (50% CT) | - | 89.0  (all CT) | 8y  91.0  (50% CT) | - | 8y  79.1  (all CT) | - | - |  |
|  |  |  |  | Low mAOL (n=187) | >0 low, ≤0 high | 92 | - | 8 | 96.3  (no CT) | - | - | 8y  94.0  (no CT) | - | - | - | - |  |
|  | DMFI | n=1,176  CT+ET vs. ET  Prosp-RCT | LN1-3  100% HR+  100% HER2- | High mAOL (n=989) | >0 low, ≤0 high | 69 | - | 31 | 96.3  (50% CT) | - | 89.3  (all CT) | 8y  92.3  (50% CT) | - | 8y  80.9  (all CT) | - | - |  |
|  |  |  |  | Low mAOL (n=187) | >0 low, ≤0 high | 92 | - | 8 | 97.5  (no CT) | - | - | 8y  95.2  (no CT) | - | - | - | - |  |
| Lopes Cardozo 2022 [39]  MINDACT | DMFI | N=201 (ultra-low)  Var ET/CT  Prosp-RCT | LN1-3  99% ER+  97% HER2- | - | >0.355 ultra-low | Ultra-low:  15 | - | - | Ultra-low:  97.4 | - | - | 8y  Ultra-low:  95.2 | - | - | - | - |  |
| Drukker 2014 [22]  VdV cohort , Netherlands | DMFS | n=144  Var ET/CT  Cohort-R | LN1-3: 74% LN4+: 26%  77% ER+ NR HER2 | Age <53y | 0.4 | 38 | - | 62 | 94.5 | - | 64.7 | 10y 78.6 25y  NE | - | 10y 54.3 25y 44.5 | 0-25 yr: HR 2.24 (1.25 to 4.00); *p*=0.01 | Y |  |
| Mook 2009 [19]  NKI and Italy | DMFS | n=241  Var ET/CT  Cohort-R | LN1-3: 100% inc micromets  79% ER+ 84% HER2- | All meno | NR | 41 | - | 59 | 98 | - | 80 | 91 | - | 76 | 0-10 yr: HR 4.13 (1.72 to 9.96); *p*=0.002  *0-10 yr: Adj HR: 2.99 (0.996 to 8.99); *p*=0.051 | Y  N* |  |
| Vliek 2017 [23]  RASTER | DRFI | N=134  Var ET/CT  Cohort-R | LN1-3  83% ER+ 85% HER2- | All ages | NR | 48 | - | 52 | 98.4 | - | 86.9 | 94.9 | - | 80.7 | 0-10 yr: Low vs high: HR 4.7 (1.3 to 16.2); *p*=0.008 | Y |  |
|  |  |  |  | All ages  High mAOL (n=109) | NR | 40 | - | 60 | 97.7 | - | 86.1 | 95.2 | - | 79.5 | 0-10 yr: Low vs high: HR 4.8 (1.1 to 21.4), *p*=0.022 | Y |  |
| **MammaPrint: DFS** | | | | | | | | | | | | | | | | | |
| Piccart 2021 [10]  MINDACT | DFS | n=1,176  CT+ET vs. ET  Prosp-RCT | LN1-3  100% HR+  100% HER2- | High mAOL (n=989) | >0 low, ≤0 high | 69 | - | 31 | 91.6  (50% CT) | - | 85.9  (all CT) | 8y  84.5  (50% CT) | - | 8y  74.5  (all CT) | - | - |  |
|  |  |  |  | Low mAOL (n=187) | >0 low, ≤0 high | 92 | - | 8 | 92.6  (no CT) | - | - | 8y  85.6  (no CT) | - | - | - | - |  |
| **MammaPrint: OS and BCSS** | | | | | | | | | | | | | | | | | |
| Piccart 2021 [10]  MINDACT | OS | n=1,176  CT+ET vs. ET  Prosp-RCT | LN1-3  100% HR+  100% HER2- | High mAOL (n=989) | >0 low, ≤0 high | 69 | - | 31 | 98.3  (50% CT) | - | 95.8  (all CT) | 8y  95.1  (50% CT) | - | 8y  89.1  (all CT) | - | - |  |
|  |  |  |  | Low mAOL (n=187) | >0 low, ≤0 high | 92 | - | 8 | 98.1  (no CT) | - | - | 8y  98.1  (no CT) | - | - | - | - |  |
| Drukker 2014 [22]  VdV cohort, Netherlands | OS | n=144  Var ET/CT  Cohort-R | LN1-3: 74% LN4+: 26%  77% ER+ NR HER2 | Age <53y | 0.4 | 38 | - | 62 | 98.2 | - | 76.9 | 10y 92.5 25y 42.2 | - | 10y 58.7 25y 47.1 | 0-25 yr: HR 1.83 (1.07 to 3.11), *p*=0.03 | Y |  |
| Jackisch 2022 (abst) [33]  Germany, PATH | OS | n=38  Var ET/CT  Cohort-R | LN+  NR | All meno (assumed) | NR | 53 | - | 47 | - | - | - | 93.3 | - | 40.4 | - | - |  |
| Mook 2009 [19]  NKI and Italy | BCSS | n=241  Var ET/CT  Cohort-R | LN1-3: 100% inc micromets  79% ER+ 84% HER2- | All meno | NR | 41 | - | 59 | 99 | - | 88 | 96 | - | 76 | 0-10 yr: HR 5.70 (2.01 to 16.23), *p*=0.001  *0-10 yr: Adj HR: 7.17 (1.81 to 28.43), *p*=0.005 | Y  Y* |  |
|  |  |  |  | All meno  High AOL (n=209) | NR | - | - | - | - | - | - | 94 | - | 76 | 0-10 yr: HR 4.12 (1.45 to 11.76); *p*=0.008 | Y |  |

*^a^The last column indicates whether each hazard ratio between test risk groups is statistically significant at the 5% level. Asterisk (*) denotes analyses adjusted for clinical factors.*

*Adj - adjusted; AOL - Adjuvant! Online; BCSS - breast cancer-specific survival; CI - confidence interval; cohort-R - cohort reanalysis; CT - chemotherapy; DFS - disease-free survival; DMFI - distant metastasis-free interval; DMFS - distant metastasis-free survival; DRFI - distant recurrence-free interval; ER - oestrogen receptor; ET - endocrine therapy; HER2 - human epidermal growth factor receptor 2; HR - hazard ratio; HR - hormone receptor; int - intermediate; LN - lymph nodes (number positive); meno - menopausal; NR - not reported; OS - overall survival; prosp - prospective; RCT - randomised controlled trial; sig - significant; var - variable; y/yr - year*

**Table D.3: Prognostic data (Prosigna)**

| **Reference**  **Study/cohort** | **Outcome** | **N, ET/CT**  **Design** | **Nodal status**  **HR, HER2** | **Meno**  **status** | **Test cut-offs** | **Distribution %** | | | **Risk 0-5yr %** | | | **Risk 0-10yr/other %** | | | **HR between test groups (95% CI)** | **^a^Sig?**  ***Adj** |  |
| --- | --- | --- | --- | --- | --- | --- | --- | --- | --- | --- | --- | --- | --- | --- | --- | --- | --- |
|  |  |  |  |  |  | **Low** | **Int** | **High** | **Low** | **Int** | **High** | **Low** | **Int** | **High** |  |  |  |
| **Prosigna: Distant recurrence, ET monotherapy** | | | | | | | | | | | | | | | | | |
| Sestak 2018 [20] 2017 [37]  TransATAC | DRFI | n=183  ET mono  RCT-R | LN1-3  100% HR+ 100% HER2- | Post-meno | NR; assume 16, 40 | 8 | 32 | 60 | 100 | 91.7 | 87.4 | 0-10y 100  5-10y 100 | 0-10y 79.3  5-10y 87.0 | 0-10y 69.3  5-10y 75.0 | 0-5yr: Int vs. high: HR 1.30 (0.47 to 3.60)  0-10yr: Int vs. high: HR 1.37 (0.69 to 2.72) HR per 1SD change: 1.58 (1.16-2.15)  *LR vs. CTS (p=0.04) and NPI (*p* = 0.09) | N  N  Y  Y, N* |  |
| Gnant 2014 [24], Filipits 2014 [25]  ABCSG-8, Austria | DMFS | n=413  ET mono  RCT-R | LN1-3: 89%  LN4+: 11%  100% ER+ 100% HER2- | Post-meno | 16, 40 | 4 | 34 | 62 | - | - | - | 0-10y 100  5-15y  100 | 0-10y 93.6  5-15y  87.0 | 0-10y 76.1  5-15y  75.0 | 5-15yr: Low risk: No events 5-15yr: Int vs. high: HR 3.15 (1.20 to 8.24); *p*=0.020  *0-10yr: Prognostic over clinical factors (*p*<0.0001)  *5-15yr: Prognostic over clinical factors (*p*=0.003) | -  Y  Y*  Y* |  |
| Laenkholm 2018 [26]  DBCG, Denmark | DRFS | n=1,395  ET mono  Cohort-R | LN1-3  100% HR+ 100% HER2- | Post-meno | Bespoke  Varies by N nodes | 26 | 28 | 46 | - | - | - | 96.5 | 88.5 | 77.9 | 0-10yr: Unadj: p<0.001  *0-10yr: Low vs. int: Adj HR 0.39 (0.20 to 0.77) *0-10yr: High vs. int: Adj HR 1.54 (1.04 to 2.26), *p*<0.001 | Y  Y*  Y* |  |
|  |  |  |  |  | 40 only | - | - | - | - | - | - | 95.2 (low to int) | | 78.1 | - | - |  |
| **Prosigna: Distant recurrence, variable ET/CT** | | | | | | | | | | | | | | | | | |
| Martin 2016 [27], Martin 2014 [28]  GEICAM 9906, Spain | DMFS | n=536  All CT+ET RCT-R | LN1-3: 64%  LN4+: 36%  100% ER+ 100% HER2- | All meno (46% post) | 18, 65 | 19 | 56 | 26 | - | - | - | 92 | 74 | 66 | 0-10yr: Low vs. int: HR 4.4 (NR)  0-10yr: Low vs. high: HR 5.8 (NR), *p*<0.0001  *Prosigna v.s EPclin + clinical factors (*p*=0.567) | -  Y  N* |  |
| **Prosigna: DFS** | | | | | | | | | | | | | | | | | |
| Pu 2020 [34]  WHEL Study | DFS | n=344  Var ET/CT  RCT-R | LN+  100% ER+ 100% HER2- | All meno | NR | 26 | 53 | 21 | - | - | - | 81 | 64 | 56 | 0-10yr: *p*=0.02 | Y |  |
| **Prosigna: OS and BCSS** | | | | | | | | | | | | | | | | | |
| Lundgren 2022 [35]  SBII:pre trial | OS | n=123  ET/ none  RCT-R | LN1-3  100% ER+ 100% HER2- | Pre-meno | 16, 40 | 2 | 42 | 57 | - | - | - | - | - | - | 0-10yr: Int vs. high: HR 1.84 (0.91–3.74); *p*=0.09  *0-10yr: Int vs. high: Adj HR 1.32 (0.61–2.88); *p*=0.48  >10yr: Int vs. high: HR 1.02 (0.54–1.93); *p*=0.96  *>10yr: Int vs. high: Adj HR 1.29 (0.66–2.53); *p*=0.46 | N  N*  N  N* |  |
| Lundgren 2022 [35]  SBII:pre trial | BCFI | n=123  ET/ none  RCT-R | LN1-3  100% ER+ 100% HER2- | Pre-meno | 16, 40 | 2 | 42 | 57 | - | - | - | - | - | - | 0-10yr: Int vs. high: HR 1.99 (1.08–3.66); *p*=0.03  *0-10yr: Int vs. high: Adj HR 1.85 (0.95–3.58); *p*=0.07  >10yr: Int vs. high: HR 1.19 (0.50–2.80); *p*=0.70  *>10yr: Int vs. high: Adj HR 1.13 (0.43–2.95); *p*=0.81 | Y  N*  N  N* |  |
| Pu 2020 [34]  WHEL Study | BCSS | n=344  Var ET/CT  RCT-R | LN+  100% ER+ 100% HER2- | All meno | NR | 26 | 53 | 21 | - | - | - | 90 | 84 | 77 | 0-10yr: *p*=0.003 | Y |  |

*^a^The last column indicates whether each hazard ratio between test risk groups is statistically significant at the 5% level. Asterisk (*) denotes analyses adjusted for clinical factors.*

*Adj - adjusted; BCFI - breast cancer-free interval; BCSS - breast cancer-specific survival; CI - confidence interval; cohort-R - cohort reanalysis; CT - chemotherapy; CTS - Clinical Treatment Score (set of clinical factors); DFS - disease-free survival; DMFS - distant metastasis-free survival; DRFI - distant recurrence-free interval; DRFS - distant recurrence-free survival; ER - oestrogen receptor; ET - endocrine therapy; HER2 - human epidermal growth factor receptor 2; HR - hazard ratio; HR - hormone receptor positive; int - intermediate; LN - lymph nodes (number positive); LR - likelihood ratio; meno - menopausal; NPI - Nottingham Prognostic Index; NR - not reported; OS - overall survival; RCT - randomised controlled trial; RCT-R - RCT reanalysis; SD - standard deviation; sig - significant; y/yr - year*

**Table D.4: Prognostic data (EPclin)**

| **Reference**  **Study/cohort** | **Outcome** | **N, ET/CT**  **Design** | **Nodal status**  **HR, HER2** | **Meno**  **Clin risk** | **Test cut-offs** | **Distribution %** | | | **Risk 0-5yr %** | | | **Risk 0-10yr/other %** | | | **HR between test groups (95% CI)** | **^a^Sig?**  ***Adj** |  |
| --- | --- | --- | --- | --- | --- | --- | --- | --- | --- | --- | --- | --- | --- | --- | --- | --- | --- |
|  |  |  |  |  |  | **Low** | **Int** | **High** | **Low** | **Int** | **High** | **Low** | **Int** | **High** |  |  |  |
| **EPclin: Distant recurrence, ET monotherapy** | | | | | | | | | | | | | | | | | |
| Sestak 2018 [20] 2017 [37]  TransATAC | DRFI | n=183  ET mono  RCT-R | LN1-3  100% HR+ 100% HER2- | Post-meno | 3.3 | 23 | - | 77 | 97.9 | - | 87.6 | 0-10y 94.4 5-10y 96.7 | 0-10y - 5-10y - | 0-10y 69.7 5-10y 76.4 | 0-5yr: High vs. low: HR 6.00 (0.80 to 44.93)  0-10yr: High vs. low: HR 6.77 (1.63 to 28.07) 0-10yr: Per 1SD change: 1.69 (1.29-2.22)  *LR vs CTS (*p*=0.20) or NPI (*p*=0.02) | N  Y  Y  N, Y* |  |
| Filipits 2019 [29]  ABCSG-6/8 | DRFR | n=453  ET mono  RCT-R | LN1-3  100% ER+ 100% HER2- | Post-meno | 3.3 | 35 | - | 65 | - | - | - | 0-10y 95.6 0-15y 84.7 5-10y 98.2 5-15y 87.0 | - | 0-10y 80.9 0-15y 75.1 5-10y 90.5 5-15y 84.0 | 0-10yr: HR 3.65 (1.73 to 7.68), *p*=0.0003  *0-10yr: Adj HR: 2.68 (1.77 to 4.08), *p*<0.0001  5-15yr: HR 3.00 (1.03 to 8.71), *p*=0.034  *5-15yr: Adj HR 3.43 (1.74 to 6.76), *p*=0.0005 | Y  Y*  Y  Y* |  |
| Sestak 2020 [36]  Lobular (from TransATAC + ABCSG-6/8) | DRFS | n=144  ET mono  RCT-R | LN1-3: 80% LN4+: 20%  100% HR+ 100% HER2- | Post-meno  Lobular | 3.3 | 26 | - | 74 | - | - | - | 93.6 | - | 68.8 | HR 3.70 (2.49 to 5.50), *p*<0.0001  *EPclin vs. clinical factors (*p*=0.0026) | Y  Y* |  |
| Constantinidou 2022 [30]  Cyprus + Notts | DRFS | n=62  ET mono  Cohort-R | LN1-3  100% ER+ 100% HER2- | Pre-meno | 3.3 | 19 | - | 81 | - | - | - | 100 | - | 75 | High vs low: *p*=0.066  *Adj HR (cont score): 2.91 (1.70 to 4.97), *p*<0.001 | N  Y* |  |
| **EPclin: Distant recurrence, variable ET/CT** | | | | | | | | | | | | | | | | | |
| Martin 2016 [27], Martin 2014 [28]  GEICAM 9906, Spain | DMFS | n=555  All CT+ET  RCT-R | LN1-3: 64%  LN4+: 36%  100% ER+ 100% HER2- | All meno (46% post) | 3.3 | 13 | - | 87 | - | - | - | 100 | - | 72 | Low vs. high: HR not estimable, *p*<0.0001  *EPclin vs. clinical factors (*p*=0.0018) | Y  Y* |  |
|  |  |  |  | Pre-meno | 3.3 | 12 | - | 88 | - | - | - | 100 | - | 70 | Low vs. high: HR NR, *p*=0.0006 | Y |  |
|  |  |  |  | Post-meno | 3.3 | 13 | - | 87 | - | - | - | 100 | - | 76 | Low vs. high: HR NR, *p*=0.0023 | Y |  |

*^a^The last column indicates whether each hazard ratio between test risk groups is statistically significant at the 5% level. Asterisk (*) denotes analyses adjusted for clinical factors.*

*Adj - adjusted; CI - confidence interval; cohort-R - cohort reanalysis; cont - continuous; CT - chemotherapy; CTS - Clinical Treatment Score (set of clinical factors); DMFS - distant metastasis-free survival; DRFI - distant recurrence-free interval; DRFR - distant recurrence-free rate; DRFS - distant recurrence-free survival; ER - oestrogen receptor; ET - endocrine therapy; HER2 - human epidermal growth factor receptor 2; HR - hazard ratio; HR - hormone receptor; int - intermediate; LN - lymph nodes (number positive); LR - likelihood ratio; meno - menopausal; NPI - Nottingham Prognostic Index; NR - not reported; RCT - randomised controlled trial; RCT-R - RCT reanalysis; SD - standard deviation; sig - significant; y/yr - year*

**Appendix E: Additional tables for observational and registry data**

**Table E.1: Observational and registry data for Oncotype DX**

| **Cohort** | **Ref** | **Nodal status**  **HR, HER2** | **Out-come** | **N**  **ET/CT** | **Meno**  **Age**  **Clin** | **Test cut-offs** | **Distribution %** | | | **% risk of outcome** | | | | **HR between test risk groups (95% CI)** | **^a^Sig?**  ***Adj** |
| --- | --- | --- | --- | --- | --- | --- | --- | --- | --- | --- | --- | --- | --- | --- | --- |
|  |  |  |  |  |  |  | **Low** | **Int** | **High** | **Low** | **Int** | **High** | |  |  |
| **Oncotype DX: Distant recurrence** | | | | | | | | | | | | | | |  |
| Clalit,  Israel | Stemmer 2017 [48] | LN1mic: 42% LN1-3: 58%  100% ER+ 100% HER2- | DRFI (0-5yr) | n=709  Var ET/CT | All meno | 18, 30 | 53 | 36 | 10 | 96.8 (7% CT) | 93.7 (40% CT) | 83.1 (86% CT) | | 0-5yr: Low vs high: HR 0.19 (0.09 to 0.40) 0-5yr: Int vs. high: HR 0.39 (0.20 to 0.79), *p*<0.001  *0-5yr: Adj HR: Low vs high: HR 0.23 (0.11 to 0.50)  *0-5yr: Adj HR: Int vs. high: HR 0.42 (0.20 to 0.86), *p*=0.001 | Y  Y  Y*  Y* |
|  |  |  |  |  |  | 11, 25 | ≤25: 81 | | 19 | 95.7 (5% CT) | 96.0  (18% CT) | 86.9 (77% CT) | | 0-5yr: *p*<0.001 | Y |
|  |  |  |  |  |  | ≤25,  26-30 |  |  |  | 96.0 (15% CT) | | 91.5 (67% CT) | | - | - |
|  |  |  |  |  |  | 18-25 |  |  |  |  | 94.4 (31% CT) |  | | - | - |
|  |  |  |  | n=109  Var ET/CT | Age <50 | 18, 30 | 48 | 37 | 16 | 96.2 (12% CT) | 100.0 (48% CT) | 64.2 (100% CT) | | 0-5yr: *p*<0.001 | Y |
|  |  |  |  | n=464  Var ET/CT | Age 50-69 | 18, 30 | 54 | 37 | 9 | 97.6 (6% CT) | 93.5 (42% CT) | 87.8 (90% CT) | | 0-5yr: *p*=0.017 | Y |
|  |  |  |  | n=136  Var ET/CT | Age ≥70 | 18, 30 | 57 | 33 | 10 | 94.7 (7% CT) | 88.7 (22% CT) | 92.9 (57% CT) | | 0-5yr: *p*=0.458 | N |
| Young Women's Breast Cancer Study | Poorvu 2020 [49] | LNmic, LN1-3  100% ER+ 100% HER2- | DRFS (0-6yr) | n=163  Var ET/CT | Age ≤40 | 18, 30 | 33 | 42 | 25 | 0-6yr: 85.9 (83% CT) | 0-6yr: 87.3 (97% CT) | 0-6yr: 62.8 (98% CT) | | 0-6yr: *p*=0.004 | Y |
|  |  |  |  |  |  | 11, 25 | 9 | 54 | 37 | 0-6yr: 92.3 (79% CT) | 0-6yr: 85.2 (92% CT) | 0-6yr: 71.3 (97% CT) | | 0-6yr: *p*=0.10 | N |
| **Oncotype DX: DFS** | | | | | | | | | | | | | | |  |
| WSG PlanB | Nitz 2017 [50] | LN1-3  100% HR+ 100% HER2- | DFS (0-5yr) | n=110  Var ET/CT | All meno | 0-10 |  |  |  | 94.4 (No CT) | - | - | | - | - |
| Red Cross Hospital, Munich, Germany | Braun 2022 [51] | LNmic: 20% LN1-3: 80%  100% HR+ 100% HER2- | DFS (0-5yr) | n=217  Var ET/CT | All meno (63% post) | ≤25, 26+ | 86 | | 14 | RS 0-25: 90.3 (19% CT) | | | 71.0 (93% CT) | - | - |
| **Oncotype DX: OS and BCSS** | | | | | | | | | | | | | | |  |
| Clalit,  Israel | Stemmer 2017 [48] | LN1mic: 42% LN1-3: 58%  100% ER+ 100% HER2- | BCSS (0-5yr) | n=709  Var ET/CT | All meno | 18, 30 | 53 | 36 | 10 | 99.5 (7% CT) | 96.6 (40% CT) | 94.3 (86% CT) | | 0-5yr: *p*<0.001 | Y |
|  |  |  |  |  |  | 11, 25 | RS≤25: 81 | | 19 | 99.1 (5% CT) | 98.8  (18% CT) | 93.5 (77% CT) | | 0-5yr: *p*<0.001 | Y |
|  |  |  |  |  |  | ≤25,  26-30 |  |  |  |  | 98.9 (15% CT) | 92.6 (67% CT) | | - | - |
|  |  |  |  |  |  | 18-25 |  |  |  |  | 97.8 (31% CT) |  | | - | - |
| SEER registry | Petkov 2016 [45] | LN1mic, LN1-3  100% HR+ 100% HER2- | BCSS (<5yr) | n=4,691  Var ET/CT | All | 18, 30 | 57 | 36 | 7 | 99.0 (23% CT) | 97.7 (47% CT) | 85.7 (75% CT) | | <5yr: High vs. low: HR 11.0 (7.8 to 15.5) <5yr: Int vs. low: HR 3.1 (2.3 to 4.3), *p*<0.001  *<5yr: Adj HR: High vs. low: HR 7.8 (5.3 to 11.6)  *<5yr: Adj HR: Int vs. low: HR 3.0 (2.1 to 4.2), *p*<0.001 | Y  Y  Y*  Y* |
|  |  |  |  | n=328  Var ET/CT | Black ethnicity | 18, 30 | 54 | 36 | 9 | 99.4 (CT NR) | 98.9 | 91.3 | | <5yr: *p*=0.4117 | N |
|  |  |  |  | n=4,021  Var ET/CT | White ethnicity | 18, 30 | 58 | 36 | 7 | 99 (CT NR) | 97.6 | 84.1 | | <5yr: *p*<0.0001 | Y |
|  |  |  |  | n=320  Var ET/CT | Other ethnicity | 18, 30 | 57 | 34 | 8 | 98.5 (CT NR) | 99.1 | 100 | | <5yr: *p*=0.8427 | N |
| SEER registry | Roberts 2017 [46] | LN1mic, LN1-3  100% HR+ 100% HER2- | BCSS (0-5yr) | n=6,483  Var ET/CT | All | 18, 30 | 58 | 35 | 7 | 98.8 (CT NR) | 97.3 | 88.5 | | 0-5yr: *p*<0.001  *0-5yr: Adj: *p*<0.001 | Y  Y* |
|  |  |  | OS (0-5yr) | n=6,483  Var ET/CT | All | 18, 30 | 58 | 35 | 7 | 92.1 (CT NR) | 90.9 | 81.7 | | 0-5yr: *p*<0.001  *0-5yr: Adj: *p*<0.001 | Y  Y* |
| SEER registry | Massarweh 2018 [47] | LN1mic, LN1-3  100% HR+ 100% HER2- | BCSS (0-5yr) | n=6,437  Var ET/CT | Women | 18, 30 | 59 | 35 | 7 | 98.8 (23% CT) | 97.3 (48% CT) | 89.2 (77% CT) | | 0-5yr: *p*<0.001 | Y |
|  |  |  |  | n=46  Var ET/CT | Men | 18, 30 | 52 | 26 | 22 | 100 (33% CT) | 100 (50% CT) | N/A (60% CT) | | 0-5yr: *p*=0.02 | Y |
|  |  |  | OS (0-5yr) | n=6,437  Var ET/CT | Women | 18, 30 | 59 | 35 | 7 | 92.2 (23% CT) | 90.8 (48% CT) | 83.2 (77% CT) | | 0-5yr: *p*<0.001 | Y |
|  |  |  |  | n=46  Var ET/CT | Men | 18, 30 | 52 | 26 | 22 | 78.9 (33% CT) | 100 (50% CT) | N/A (60% CT) | | 0-5yr: *p*=0.002 | Y |
| NCDB | Ibraheem 2020 [40] | LN1-3  100% HR+ 100% HER2- | OS (0-5yr) | n=25,029  Var ET/CT | All meno | 11, 25 | 24 | 64 | 13 | - | - | - | | 0-5yr:  Int vs low: HR 1.15 (0.97 to 1.36) High vs low: HR 2.94 (2.43 to 3.56) Per 10-RS: HR 1.38 (1.31 to 1.44) | N  Y  Y |
| NCDB | Ibraheem 2019 [41] | LN1-3: 97% LN4-9: 3% | OS (0-5yr) | n=13,163  Var ET/CT | All meno | 11, 25 | - | - | - | - | - | - | | 0-5yr:  RS 18-25 vs 11-17: HR 1.20 (1.07-1.35), *p*<0.001  *RS 18-25 vs 11-17: Adj HR 1.15 (1.03-1.29), *p*<0.001 RS 26-30 vs 11-17: HR 1.91 (1.65-2.22), *p*<0.001  *RS 26-30 vs 11-17: Adj HR 1.62 (1.38-1.89), *p*<0.001 | Y  Y*  Y  Y* |
| NCDB | Nash 2023 [42] | LN1-3  100% HR+ 100% HER2- | OS (NR, med FU 5.5yr) | N=4,124  Var ET/CT | Age 40-50 | 11, 25 | - | - | - | - | - | - | | *RS 26-30 vs 0-25: Adj HR 2.29 (1.49 to 4.86) *RS 31-50 vs. 0-25: Adj HR 3.70 (2.03 to 6.75) *RS 51-100 vs 0-25: Adj HR 2.31 (0.78–6.86) *p*<0.001 | Y*  Y*  N* |
| NCDB | Weiser 2022 [43] | LN1-3  100% HR+ 100% HER2- | OS (0-5yr) | n=2,691  Var ET/CT | Lobular | 11, 25 | - | - | - | 95.5 | 95.5 | 83.8 | | 0-5yr: *p*=0.0004  *Adj: sig | Y  Y* |
| NCDB | Weiser 2021 [44] | LN1-3  100% HR+ 100% HER2- | OS (0-5yr) | n=28,591  Var ET/CT | All | ≤25 | - | - | - | - | - | - | | *0-5yr: RS 18-25 vs. RS 12-17: Adj HR 1.30 (1.00 to 1.68) | Y* |

*^a^The last column indicates whether each hazard ratio between test risk groups is statistically significant at the 5% level. Asterisk (*) denotes analyses adjusted for clinical factors.*

*Adj - adjusted; BCSS - breast cancer-specific survival; CI - confidence interval; CT - chemotherapy; DFS - disease-free survival; DRFI - distant recurrence-free interval; DRFS - distant recurrence-free survival; ER - oestrogen receptor; ET - endocrine therapy; HER2 - human epidermal growth factor receptor 2; HR - hazard ratio; HR - hormone receptor; int - intermediate; LN - lymph nodes (number positive); meno - menopausal; NR - not reported; OS - overall survival; sig - significant; var - variable; y/yr - year*

**Appendix F: Additional tables for prediction of chemotherapy benefit from observational and registry data**

**Table F.1: Observational and registry data:** **prediction of chemotherapy benefit for Oncotype DX**

| **Cohort** | **Ref** | **Nodal status**  **HR, HER2** | **Out-come** | **N** | **Meno**  **Age**  **Clin** | **Test cut-offs** | **% risk of outcome** | | | | | | | | | | **Abs diff CT v no CT** | | | | **HR for CT vs. no CT (95% CI)** | | | **Inter-action** | **^a^Pred**  ***Adj** |
| --- | --- | --- | --- | --- | --- | --- | --- | --- | --- | --- | --- | --- | --- | --- | --- | --- | --- | --- | --- | --- | --- | --- | --- | --- | --- |
|  |  |  |  |  |  |  | **Low** | | | | **Int** | | | | **High** | | **Low** | **Int** | | **High** | **Low** | **Int** | **High** |  |  |
|  |  |  |  |  |  |  | **CT** | | **No** | | **CT** | | **No** | | **CT** | **No** |  |  |  |  |  |  |  |  |  |
| **Oncotype DX: Observational: Distant recurrence** | | | | | | | | | | | | | | | | | | | | | | | | | |
| Clalit, Israel | Stemmer 2017 [48] | LN1mic: 42% LN1-3: 58%  100% ER+ 100% HER2- | DRFI 0-5yr | n=709 | All meno | 18, 30 | 92.3 | 97.1 | | 99 | | 90.3 | | 82 | | 90 | -4.8 | 8.7 | -8.0 | | *p*=0.245 | *p*=0.019 | - | - | - |
|  |  |  |  |  |  | 11, 25 | 83.3 | 96.3 | | 98.8 | | 95.4 | | 97.5 | | 79.7 | -13.0 | 3.4 | 17.8 | | - | - | *p*=0.017 | - | - |
|  |  |  |  |  |  | All ≤25 | - | - | | 97.7 | | 95.6 | | - | | - | 2.1 | | - | | *p*=0.521 | | - | - | - |
|  |  |  |  |  |  | 18-25 | - | - | | 100 | | 91.8 | | - | | - | - | 8.2 | - | | - | *p*=0.058 | - | - | - |
|  | Rotem 2022 (abst) [52] | LN+  100% ER+  100% HER2- | DRFS 0-7yr | n=140 | All meno | All  26-30 | - | - | | - | | - | | 89.4 | | 78.0 | - | - | 11.4 | | - | - | Not sig | - | - |
| **Oncotype DX: Observational: BCSS and OS** | | | | | | | | | | | | | | | | | | | | | | | | | |
| Clalit, Israel | Stemmer 2017 [48] | LN1mic: 42% LN1-3: 58%  100% ER+ 100% HER2- | BCSS 0-5yr | n=709 | All meno | 18, 30 | 100.0 | 99.4 | | 98.9 | | 95.1 | | 93.4 | | 100 | 0.6 | 3.8 | -6.6 | | - | - | - | - | - |
|  |  |  |  |  |  | 11, 25 | 100.0 | 99.1 | | 100.0 | | 98.6 | | 97.1 | | 84.0 | 0.9 | 1.4 | 13.1 | | - | - | - | - | - |
|  |  |  |  |  |  | All ≤25 | - | - | | 100.0 | | 98.7 | | - | | - | 1.3 | | - | | - | - | - | - | - |
|  |  |  |  |  |  | 18-25 | - | - | | 100.0 | | 96.8 | | - | | - | - | 3.2 | - | | - | - | - | - | - |
|  | Rotem 2022 (abst) [52] | LN+  100% ER+  100% HER2- | BCSS 0-7yr | n=140 | All meno | 26-30 | - | - | | - | | - | | 98.7 | | 93.8 | - | - | 4.9 | | - | - | *p*=0.024 | - | - |
| SEER | Petkov 2020 (abst) [53] | LN1mic-LN3  100% HR+ 100% HER2- | BCSS 0-5yr | n=2,588 | Age≤50 | 0-10 | 100 | 100 | | - | | - | | - | | - | 0 | - | - | | - | - | - | - | - |
|  |  |  |  |  |  | 11-15 | - | - | | 97.7 | | 99.5 | | - | | - | - | -1.8 | - | | - | - | - | - | - |
|  |  |  |  |  |  | 16-20 | - | - | | 98.4 | | 98.7 | | - | | - | - | -0.3 | - | | - | - | - | - | - |
|  |  |  |  |  |  | 21-25 | - | - | | 98.8 | | 98.4 | | - | | - | - | 0.4 | - | | - | - | - | - | - |
|  |  |  |  |  |  | 26-100 | - | - | | - | | - | | 93.9 | | 95.6 | - | - | -1.7 | | - | - | - |  |  |
| Clalit, Israel | Rotem 2022 (abst) [52] | LN+  100% ER+  100% HER2- | OS 0-7yr | n=140 | All meno | 26-30 | - | - | | - | | - | | 96.3 | | 93.8 | - | - | 2.5 | | - | - | Not sig | - | - |
| NCDB | Abel 2022 [54] | LN1-3  100% HR+ 100% HER2- | OS 0-5yr | n=21,370 | Ductal | All ≤25 | - | - | | - | | - | | - | | - | - | - | - | | *p*=0.278 | | - | - | - |
|  |  |  |  | n=6,356 | Lobular | All ≤25 | - | - | | - | | - | | - | | - | - | - | - | | *p*=0.532 | | - | - | - |
|  |  |  |  | n=4,251 | Age<50 Ductal | All ≤25 | - | - | | - | | - | | - | | - | - | - | - | | Unadj: 0.44 (0.22 to 0.86), *p*=0.016 | | - | - | - |
|  |  |  |  | n=1,062 | Age<50 Lobular | All ≤25 | - | - | | - | | - | | - | | - | - | - | - | | Unadj: 0.54 (0.14 to 2.18), *p*=0.39 | | - | - | - |
| NCDB (cont) | Cao 2022 (abst) [55] | LN1-3  100% ER+ 100% HER2- | OS NR | n=28,427 | Age≤50 | All  20-25 | - | - | | - | | - | | - | | - | - | - | - | | - | Unadj: 0.334 (NR), *p*=0.002 | - | - | - |
|  |  |  |  |  | Age>50 | All  20-25 | - | - | | - | | - | | - | | - | - | - | - | | - | Unadj: 0.521 (NR), *p*=0.019 | - | - | - |
| NCDB (cont) | Ibraheem 2019 [41] | LN1-3: 97% LN4-9: 3%  100% HR+ 100% HER2- | OS 0-5yr | n=13,163 | All meno | 11-17 | - | - | | 97.7 | | 96.5 | | - | | - | - | 1.2 | - | | - | Adj: 0.63 (0.40 to 0.99), *p*=0.044  Threshold: RS >13 sig CT benefit | - | - | - |
|  |  |  |  |  |  | 18-25 | - | - | | 96.0 | | 92.7 | | - | | - | - | 3.3 | - | | - | Adj: 0.53 (0.37 to 0.76), *p*=0.001 | - | - | - |
|  |  |  |  |  |  | 26-30 |  |  | |  | |  | | 92.2 | | 85.5 |  |  | 6.7 | |  |  | Adj: 0.50 (0.28 to 0.89), *p*=0.018 |  |  |
|  |  |  |  | n=3,101 | Age≤50 | All  11-25 | - | - | | - | | - | | - | | - | - | - | - | | - | Adj: 0.68 (0.35 to 1.32), *p*=0.25 | - | - | - |
|  |  |  |  | n=8,886 | Age>50 | All  11-25 | - | - | | - | | - | | - | | - | - | - | - | | - | Adj: 0.64 (0.47 to 0.86), *p*=0.004 | - | - | - |
| NCDB (cont) | Iorgulescu 2019 [56] | LN1-3  100% ER+ 100% HER2- | OS 0-5yr | n=2,735 | All meno | 18, 30 | 93 | 92 | | 93.2 | | 85.7 | | 92.4 | | 66.9 | 1.0 | 7.5 | 25.5 | | Unadj: p=0.27 Adj: 0.81 (0.33 to 1.98, *p*=0.64 | Unadj: p=0.02 Adj: 0.67 (0.35 to 1.27), *p*=0.22 | Unadj: p<0.001 Adj: 0.24 (0.13 to 0.47), *p*<0.001 | - | - |
| NCDB (cont) | Kumar 2023 (abst) [57] | LN1-3: >90% LN4+: <10%  100% HR+ 100% HER2- | OS 0-10yr | n=8,628 | Age≤50 | 0-11 | - | - | | - | | - | | - | | - | - | - | - | | Adj: 0.56 (0.22 to 1.42) | - | - | - | - |
|  |  |  |  |  |  | 12-25 | - | - | | - | | - | | - | | - | - | - | - | | - | Adj: 0.55 (0.38 to 0.80) | - |  |  |
|  |  |  |  |  |  | All ≤25 | - | - | | 93.0 | | 91.0 | | - | | - | 2.0 | | - | | Unadj: 0.60 (0.48 to 0.75), *p*<0.0001 Adj: 0.54 (0.39 to 0.76), *p*=0.0004 | | - | - | - |
|  |  |  |  | n=8,628 | Age 18-40 | All ≤25 | - | - | | 86.0 | | 82.8 | | - | | - | 3.2 | | - | | Adj: 0.43 (0.22 to 0.85) | | - | - | - |
|  |  |  |  | n=8,628 | Age 40-50 | All ≤25 | - | - | | 94.7 | | 92.2 | | - | | - | 2.5 | | - | | Adj: 0.59 (0.39 to 0.87) | | - | - | - |
| NCDB (cont) | Nash 2023 [42] | LN1-3  100% HR+ 100% HER2- | OS NR, med FU 5.5yr | N=4,124 | Age 40-50 | All ≤25 | - | - | | - | | - | | - | | - | - | - | - | | Unadj: *p*=0.41 Adj: 0.72 (0.47 to 1.12), *p*=0.15 | | - | - | - |
|  |  |  |  |  |  | 25-30 | - | - | | - | | - | | - | | - | - | - | - | | - | | Unadj: *p*=0.28 |  |  |
|  |  |  |  |  |  | 31-50 | - | - | | - | | - | | - | | - | - | - | - | | - | | Unadj: p=0.002 Adj: 0.29 (0.10 to 0.85), *p*=0.02 |  |  |
|  |  |  |  |  |  | >50 | - | - | | - | | - | | - | | - | - | - | - | | - | | Not sig (few events) |  |  |
| NCDB (cont) | Weiser 2022 [43] | LN1-3  100% HR+ 100% HER2- | OS 0-5yr | n=16,646 | All meno  Ductal | 11-25 | - | - | | 96.7 | | 95.1 | | - | | - | - | 1.6 | - | | - | Unadj: *p*=0.004 Adj: non-sig | - | - | - |
|  |  |  |  | NR | Age<50  Ductal | All ≤25 | - | - | | - | | - | | - | | - | - | - | - | | Adj: 2.32 (1.19 to 4.49) | | - | - | - |
|  |  |  |  | NR | Age 50-75  Ductal | All ≤25 | - | - | | - | | - | | - | | - | - | - | - | | Adj: 1.12 (0.86 to 1.46) | | - | - | - |
|  |  |  |  | n=2,691 | All meno  Lobular | 0-10 | 94.7 | 95.7 | | - | | - | | - | | - | -1.0 | - | - | | Unadj: *p*=0.888 Adj: non-sig | - | - | - | - |
|  |  |  |  |  |  | 11-25 | - | - | | 96.6 | | 94.9 | | - | | - | - | 1.7 | - | | - | Unadj: *p*=0.381 Adj: non-sig | - |  |  |
| NCDB (cont) | Weiser 2021 [44] | LN1-3  100% HR+ 100% HER2- | OS 0-5yr | n=28,591 | All meno | All ≤25 | - | - | | 96.6 | | 93.2 | | - | | - | 3.4 | | - | | Unadj: p<0.001 Adj: 1.63 (1.28 to 2.07) | | - | - | - |
|  |  |  |  | NR | Age≤50 | All ≤25 | - | - | | - | | - | | - | | - | 1.4 | | - | | Adj: 1.88 (1.05 to 3.37), *p*=0.032 | | - | - | - |
|  |  |  |  |  |  | 12-17 | - | - | | - | | - | | - | | - | - | 1.3 | - | | - | Adj: 2.49 (0.80 to 7.76) | - | - | - |
|  |  |  |  |  |  | 18-25 | - | - | | - | | - | | - | | - | - | 4.4 | - | | - | Adj: 3.30 (1.38 to 7.84) | - | - | - |
|  |  |  |  | NR | Age 51-70 | All ≤25 | - | - | | - | | - | | - | | - | 1.6 | | - | | Adj: 1.49 (1.12 to 1.97), *p*=0.006 | | - | - | - |
|  |  |  |  |  |  | 12-17 | - | - | | - | | - | | - | | - | - | 3.6 | - | | - | Adj: 2.80 (1.45 to 5.24) | - | - | - |
|  |  |  |  |  |  | 18-25 | - | - | | - | | - | | - | | - | - | 3.2 | - | | - | Adj: 1.37 (0.92–2.05) | - | - | - |
|  |  |  |  | NR | Age>70 | All ≤25 | - | - | | - | | - | | - | | - | - | - | - | | Adj: 1.1 (0.68 to 1.78), *p*=0.69 | | - | - | - |
|  |  |  |  | NR | Age≤70 | 0-10 | - | - | | - | | - | | - | | - | - | - | - | | *p*=0.44 | - | - |  |  |
|  |  |  |  |  |  | 12-25 | - | - | | - | | - | | - | | - | - | 3.0 | - | | - | Adj: 1.91 (1.42 to 2.57) | - | - | - |
|  |  |  |  |  |  | 12-17 | - | - | | - | | - | | - | | - | - | 3.4 | - | | - | Adj: 3.04 (1.78 to 5.21), *p*<0.001 | - | - | - |
|  |  |  |  |  |  | 18-25 | - | - | | - | | - | | - | | - | - | 3.8 | - | | - | Adj: 2.02 (1.42 to 2.87), *p*<0.001 | - | - | - |

*^a^The Last column indicates whether interaction test (between risk group and CT use) indicates a significant predictive effect for CT benefit at the 5% level. Asterisk (*) denotes interaction adjusted for clinical factors.*

*Abs diff - absolute difference; adj - adjusted; BCSS - breast cancer-specific survival; CI - confidence interval; CT - chemotherapy; DRFI - distant recurrence-free interval; DRFS - distant recurrence-free survival; ER - oestrogen receptor; HER2 - human epidermal growth factor receptor 2; HR - hazard ratio; HR - hormone receptor; int - intermediate; LN - lymph nodes (number positive); meno - menopausal; NR - not reported; OS - overall survival; prosp - prospective; pred - predictive of CT benefit; RCT - randomised controlled trial; RS – Recurrence Score (Oncotype DX); sig - significant; unadj - unadjusted; yr - year*

**Appendix G: Additional tables for decision impact**

**Table G.1: Decision impact: Oncotype DX (not split by test risk group)**

| **Ref**  **Country** | **Study, setting**  **Years** | **HR, HER2** | **Nodal status**  **Clinical risk** | **Recom/ decision** | **Meno status** | **Test group** | **RxP** | **N pts** | **No CT** | **No CT to CT** | **CT** | **CT to no CT** | **Pre-test CT** | **Post-test CT** | **Net change CT** |
| --- | --- | --- | --- | --- | --- | --- | --- | --- | --- | --- | --- | --- | --- | --- | --- |
| Battisti 2019 (abst) [58]  **UK** | PONDx; 30 centres  2017-2018 | ER+ HER2- | LN1-3 | R-R | All (65% post) | All RS | - | 567 | - | - | - | - | 371 (65%) | 162 (29%) | -209 (-37%) |
|  |  |  |  | R-D | All (65% post) | All RS | - | 567 | - | - | - | - | 371 (65%) | 140 (25%) | -231 (-41%) |
| Holt 2024 [59, 70]  **UK** | 14 centres  2017-2022 | ER+ HER2- | LN1-3 | R-D | All (77% post) | All RS | - | 664 | 117 (18%) | 17 (3%) | 171 (26%) | 359 (54%) | 530 (80%) | 188 (28%) | -342 (-52%) |
|  |  |  |  |  | Pre-meno | All RS | - | 152 | 23 (15%) | 6 (4%) | 65 (43%) | 58 (38%) | 123 (81%) | 71 (47%) | -52 (-34%) |
|  |  |  |  |  | Post-meno | All RS | - | 512 | 94 (18%) | 11 (2%) | 106 (21%) | 301 (59%) | 407 (79%) | 117 (23%) | -290 (-57%) |
| Loncaster 2017 [60]  **UK** | Greater Manchester (NR centres)  2012-2015 | ER+ HER2- | LN+  CT indicated.  Post-test decision based on RS | R-D | Post-meno | All RS | - | 65 | 0 (0%) | 0 (0%) | 20 (31%) | 45 (69%) | 65 (100%) | 20 (31%) | -45 (-69%) |
| Malam 2022 [61]  **UK** | Norfolk and Norwich (1 centre)  2014-2020 | ER+ HER2- | LN1-3  Post-test decision based on RS | R-R | All meno | All RS | - | 69 | 36 (52%) | 1 (1.4%) | 8 (12%) | 24 (35%) | 32 (46%) | 9 (13%) | -19 (-28%) |
| Nanda 2021 (abst) [62]  **UK** | Oxford + Swansea (2 centres)  2013-2019 | ER+ HER2- | LN1-3 (inc micromets)  CT indicated | R-R | All meno | All RS | - | 173 | 0 (0%) | 0 (0%) | 44 (25%) | 129 (75%) | 173 (100%) | 44 (25%) | -129 (-75%) |
| Eiermann 2013 [69]  **Germany** | 15 centres  2010-2011 | ER+ HER2- | LN1-3 | R-R | All meno | All RS | - | 122 | 18 (15%) | 12 (10%) | 58 (46%) | 34 (28%) | 92 (75%) | 70 (57%) | -22 (-18%) |
|  |  |  |  | R-D | All meno | All RS | - | 122 | - | - | - | - | 92 (75%) | 57 (47%) | -35 (-29%) |
| Cognetti 2021 [63]  **Italy** | PONDx; 27 centres  2016-2017 | ER+ HER2- | LN1-3 | R-R | All (55% post) | All RS | - | 414 | - | - | - | - | 258 (62%) | 110 (28%) | -148 (-55%) |
| Dieci 2019 [65]  **Italy** | ROXANE; 9 centres  2017-2018 | HR+ HER2- | LN1-3  94% high clin risk (mAOL) | R-R | All (55% post) | All RS | - | 99 | 42 (42%) | 3 (3%) | 24 (24%) | 30 (30%) | 54 (55%) | 27 (27%) | -27 (-27%) |
| Dieci 2018 [64]  **Italy** | Breast DX, 9 centres  2014-2016 | ER+ HER2- | LN1-3  Int clin risk | R-R | All (55% post) | All RS | - | 126 | 49 (39%) | 5 (4%) | 52 (41%) | 20 (16%) | 72 (57%) | 57 (45%) | -15 (-12%) |
|  |  |  |  | R-D | All (55% post) | All RS | - | 126 | - | - | - | - | 72 (57%) | 54 (43%) | -18 (-14%) |
| Zambelli 2020 [68]  **Italy** | BONDX (4 centres, Lombardy)  2017-2018 | ER+ HER2- | LN1-3  Int clin risk | R-R | All meno | All RS | - | 127 | 79 (62%) | 0 (0%) | 25 (20%) | 23 (18%) | 48 (38%) | 25 (20%) | -23 (-18%) |
| Fernandez-Perez 2021 (abst) [66]  **Spain** | 9 centres (Galicia)  2013-2018 | HR+ HER2- | LN1-3 (inc micromets) | R-R | All (50% post) | All RS | - | 229 | - | - | - | - | 159 (69%) | 59 (26%) | -100 (-44%) |
| Llombart-Cussac 2023 [67]  **Spain** | KARMA Dx (8 centres)  2016-2017 | ER+ HER2- | LN1-3  High clin risk  CT indicated | R-R | All meno | All RS | - | 150 | 0 (0%) | 0 (0%) | 41 (27%) | 109 (73%) | 150 (100%) | 41 (27%) | -109 (-73%) |

*CT - chemotherapy; D - decision; ER - oestrogen receptor; HER2 - human epidermal growth factor receptor 2; HR - hormone receptor positive; LN - lymph nodes (number positive); meno - menopausal; NR - not reported; Pre/post-RxP - Pre/post publication of RxPONDER; R - recommendation; RS - Recurrence Score (Oncotype DX).*

**Table G.2: Decision impact: Oncotype DX (results by test risk group)**

| **Ref**  **Country** | **Study, setting**  **Years** | **HR, HER2** | **Nodal status**  **Clinical risk** | **Recom/ decision** | **Meno status** | **Test group** | **RxP** | **N pts** | **No CT** | **No CT to CT** | **CT** | **CT to no CT** | **Pre-test CT** | **Post-test CT** | **Net change CT** |
| --- | --- | --- | --- | --- | --- | --- | --- | --- | --- | --- | --- | --- | --- | --- | --- |
| **Oncotype DX: Cut-offs of RS 18 and 30** | | | | | | | | | | | | | | | |
| Holt 2024 [59, 70]  **UK** | 14 centres  2017-2022 | ER+ HER2- | LN1-3 | R-D | All meno | RS 0-17 | - | 400 | 95 (24%) | 3 (1%) | 28 (7%) | 274 (69%) | 302 (76%) | 31 (8%) | -271 (-68%) |
|  |  |  |  |  |  | RS 18-30 | - | 204 | 20 (10%) | 12 (6%) | 88 (43%) | 84 (41%) | 172 (84%) | 100 (49%) | -72 (-35%) |
|  |  |  |  |  |  | RS >30 | - | 58 | 0 (0%) | 2 (3%) | 55 (95%) | 1 (1.7%) | 56 (97%) | 57 (98%) | +1 (+1.7%) |
| Loncaster 2017 [60]  **UK** | Greater Manchester (NR centres)  2012-2015 | ER+ HER2- | LN+  CT indicated.  Post-test decision based on RS | R-D | Post-meno | RS 0-17 | - | 40 | 0 (0%) | 0 (0%) | 3 (8%) | 37 (93%) | 40 (100%) | 3 (8%) | -37 (-93%) |
|  |  |  |  |  |  | RS 18-30 | - | 19 | 0 (0%) | 0 (0%) | 12 (63%) | 7 (37%) | 19 (100%) | 12 (63%) | -7 (-37%) |
|  |  |  |  |  |  | RS >30 | - | 6 | 0 (0%) | 0 (0%) | 5 (83%) | 1 (17%) | 6 (100%) | 5 (83%) | -1 (-17%) |
| Eiermann 2013 [69]  **Germany** | 15 centres  2010-2011 | ER+ HER2- | LN1-3 | R-R | All meno | RS 0-17 | - | 67 | - | 2 (3%) | - | 30 (45%) | - | - | - |
|  |  |  |  |  |  | RS 18-30 | - | 44 | - | 8 (18%) | - | 4 (9%) | - | - | - |
|  |  |  |  |  |  | RS 31+ | - | 11 | - | 2 (18%) | - | 0 (0%) | - | - | - |
| Llombart-Cussac 2023 [67]  **Spain** | KARMA Dx (8 centres)  2016-2017 | ER+ HER2- | LN1-3  High clin risk  CT indicated | R-R | All meno | RS 0-17 | - | 86 | 0 (0%) | 0 (0%) | 8 (9%) | 78 (91%) | 86 (100%) | 8 (9%) | -78 (-91%) |
|  |  |  |  |  |  | RS 18-30 | - | 57 | 0 (0%) | 0 (0%) | 26 (46%) | 31 (54%) | 57 (100%) | 26 (46%) | -31 (-54%) |
|  |  |  |  |  |  | RS 31-100 | - | 7 | 0 (0%) | 0 (0%) | 7 (100%) | 0 (0%) | 7 (100%) | 7 (100%) | No change |
| Zambelli 2020 [68]  **Italy** | BONDX (4 centres, Lombardy)  2017-2018 | ER+ HER2- | LN1-3  Int clin risk | R-R | All meno | RS 0-17 | - | 71 | 56 (79%) | 0 (0%) | 1 (1%) | 14 (20%) | 15 (21%) | 1 (1%) | -14 (-20%) |
|  |  |  |  |  |  | RS 18-30 | - | 48 | 23 (48%) | 0 (0%) | 16 (33%) | 9 (19%) | 25 (52%) | 16 (33%) | -9 (-19%) |
|  |  |  |  |  |  | RS 31-100 | - | 8 | 0 (0%) | 0 (0%) | 8 (100%) | 0 (0%) | 8 (100%) | 8 (100%) | No change |
| **Oncotype DX: Cut-offs of RS 11 and 25** | | | | | | | | | | | | | | | |
| Holt 2024 [59, 70]  **UK** | 14 centres  2017-2022 | ER+ HER2- | LN1-3 | R-D | All meno | RS 0-13 | - | 261 | 68 (26%) | 2 (1%) | 13 (5%) | 178 (68%) | 191 (73%) | 15 (6%) | -176 (-67%) |
|  |  |  |  |  |  | RS 14-25 | - | 305 | 48 (16%) | 7 (2%) | 72 (24%) | 178 (58%) | 250 (82%) | 79 (26%) | -171 (-56%) |
|  |  |  |  |  |  | RS 26-100 | - | 98 | 1 (1%) | 8 (8%) | 86 (88%) | 3 (3%) | 89 (91%) | 94 (96%) | +5 (+5%) |
|  |  |  |  |  | Pre-meno | RS 0-25 | - | 127 | 23 (18%) | 4 (3%) | 43 (34%) | 57 (45%) | 100 (79%) | 47 (37%) | -53 (-42%) |
|  |  |  |  |  |  | RS 26-100 | - | 25 | 0 (0%) | 2 (8%) | 22 (88%) | 1 (4%) | 23 (92%) | 24 (96%) | +1 (+4%) |
|  |  |  |  |  | Post-meno | RS 0-25 | - | 439 | 93 (21%) | 5 (1%) | 42 (10%) | 299 (68%) | 341 (78%) | 47 (11%) | -294 (-67%) |
|  |  |  |  |  |  |  | Pre-RxP | 292 | 57 (20%) | 1 (0.3%) | 40 (14%) | 194 (66%) | 234 (80%) | 41 (14%) | -193 (-66%) |
|  |  |  |  |  |  |  | Post-RxP | 147 | 36 (24%) | 4 (3%) | 2 (1%) | 105 (71%) | 107 (73%) | 6 (4%) | -101 (-69%) |
|  |  |  |  |  |  | RS 26-100 | - | 73 | 1 (1%) | 6 (8%) | 64 (88%) | 2 (3%) | 66 (90%) | 70 (96%) | +4 (+5%) |
|  |  |  |  |  |  |  | Pre-RxP | 44 | 1 (2%) | 5 (11%) | 36 (82%) | 2 (5%) | 38 (86%) | 41 (93%) | +3 (+7%) |
|  |  |  |  |  |  |  | Post-RxP | 29 | 0 (0%) | 1 (3%) | 28 (97%) | 0 (0%) | 28 (97%) | 29 (100%) | +1 (+3%) |
| Dieci 2019 [65]  **Italy** | ROXANE; 9 centres  2017-2018 | HR+ HER2- | LN1-3  Most high clin risk (mAOL) | R-R | All (55% post) | RS<11 | - | 31 | - | - | - | - | 19 (61%) | 3 (10%) | -16 (-52%) |
|  |  |  |  |  |  | RS 11-25 | - | 61 | - | - | - | - | 28 (46%) | 17 (28%) | -11 (-18%) |
|  |  |  |  |  |  | RS 11-17 | - | NR | - | - | - | - | - (49%) | - (19.5%) | NR (-29.5%) |
|  |  |  |  |  |  | RS 18-25 | - | NR | - | - | - | - | - (40%) | - (45%) | NR (+5%) |
|  |  |  |  |  |  | RS ≥26 | - | 7 | - | - | - | - | 7 (100%) | 7 (100%) | No change |

*CT - chemotherapy; D - decision; ER - oestrogen receptor; HER2 - human epidermal growth factor receptor 2; HR - hormone receptor positive; LN - lymph nodes (number positive); meno - menopausal; NR - not reported; Pre/post-RxP - Pre/post publication of RxPONDER; R - recommendation; RS - Recurrence Score (Oncotype DX)*
